# Vaccination Against Whipworm: Identification of Potential Immunogenic Proteins in *Trichuris muris* Excretory/Secretory Material

**DOI:** 10.1038/s41598-018-22783-y

**Published:** 2018-03-14

**Authors:** Rebecca K. Shears, Allison J. Bancroft, Catherine Sharpe, Richard K. Grencis, David J. Thornton

**Affiliations:** Wellcome Trust Centre for Cell-Matrix Research and Manchester Immunology Group, School of Biological Sciences, Faculty of Biology, Medicine and Health, Manchester Academic Health Sciences Centre, University of Manchester, Manchester, M13 9PT England

## Abstract

*Trichuris trichiura* (whipworm) is one of the four major soil-transmitted helminth infections of man, affecting an estimated 465 million people worldwide. An effective vaccine that induces long-lasting protective immunity against *T*. *trichiura* would alleviate the morbidity associated with this intestinal-dwelling parasite, however the lack of known host protective antigens has hindered vaccine development. Here, we show that vaccination with ES products stimulates long-lasting protection against chronic infection in male C57BL/6 mice. We also provide a framework for the identification of immunogenic proteins within *T*. *muris* ES, and identify eleven candidates with direct homologues in *T*. *trichiura* that warrant further study. Given the extensive homology between *T*. *muris* and *T*. *trichiura* at both the genomic and transcriptomic levels, this work has the potential to advance vaccine design for *T*. *trichiura*.

## Introduction

Soil-transmitted helminths (STHs) are a huge source of morbidity in humans and domestic animals^1^. The four major human STH species are *T*. *trichiura* (whipworm), *Ascaris lumbricoides* and the hookworms, *Necator americanus* and *Ancyclostoma duodenale*^2^. Over a billion people worldwide are infected with one or more of these parasites, which reside within the gastrointestinal tract of their host, costing an estimated 4.5 to 39 million disability adjusted life years per annum^3,4^. STH infections can be associated with anaemia, growth stunting and cognitive impairment, especially in children^2^. For *T*. *trichiura*, which resides primarily in the caecum, heavy worm burdens are associated with *Trichuris* dysentery syndrome, symptoms of which include stomach pain, diarrhoea and in extreme cases, rectal prolapse^2,5^. A combination of anti-helminthic drugs, sanitation improvements and prophylactic vaccines are predicted to reduce the morbidity associated with these infections^6^. However, a recent meta-analysis showed that the benzamidazole drugs currently used to treat STH infections have poor efficacy against *T*. *trichiura*, and there are reports of drug resistance arising within parasite populations in Vietnam and Zanzibar^7–9^. In addition, there are no commercially available vaccines against human STH species, and few against their veterinary counterparts^10^.

The naturally-occurring murine whipworm, *T*. *muris*, has been used for over 50 years as a model for *T*. *trichiura*^11^. These parasites share extensive homology at the genomic and transcriptomic levels, and the immune responses associated with both acute and chronic infection have been well studied using the *T*. *muris* mouse model^12,13^. Infection dose can influence the relative resistance/susceptibility of mice to *T*. *muris*. Most standard laboratory strains are capable of expelling a high dose infection (200 eggs), while a low dose infection (10–25 eggs) promotes an IFN-γ dominated CD4 + T helper cell type 1 (Th1) response, leading to chronic infection, which is more reflective of natural challenge^14^. During acute infection, worm expulsion is driven by a Th2 response, and the Th2 cytokines, IL-13 and IL-9, are known to stimulate effector mechanisms that drive worm expulsion^13^. These effector mechanisms include increasing mucus production and epithelial turnover in the caecum (IL-13), and inducing intestinal hypercontractility (IL-9)^15–17^. Natural immunity is acquired during acute infection, and mice are protected against all subsequent infections (both high and low dose)^14^.

Vaccination with *T*. *muris* ES has previously been shown to protect naturally susceptible AKR mice against a subsequent high dose infection and promote earlier expulsion of an acute infection in NIH mice^18–20^. However, relatively little progress has been made towards identifying the parasite antigens responsible for triggering expulsion, and crucially the potential for ES vaccinations to protect mice against a low dose infection, which naturally primes for chronicity, has yet to be investigated. The work described here shows that vaccination with *T*. *muris* ES proteins stimulates long-lasting protection against a subsequent low dose infection in C57BL/6 mice. Potential immunogenic candidates within *T*. *muris ES* were identified using gel filtration chromatography and mass spectrometry methods in combination with a measurement of T cell cytokine production. Several potential immunogenic proteins were identified, all of which have direct homologues in *T*. *trichiura*.

## Results

### Narrowing down the search for protective antigens

**Figure 1:**
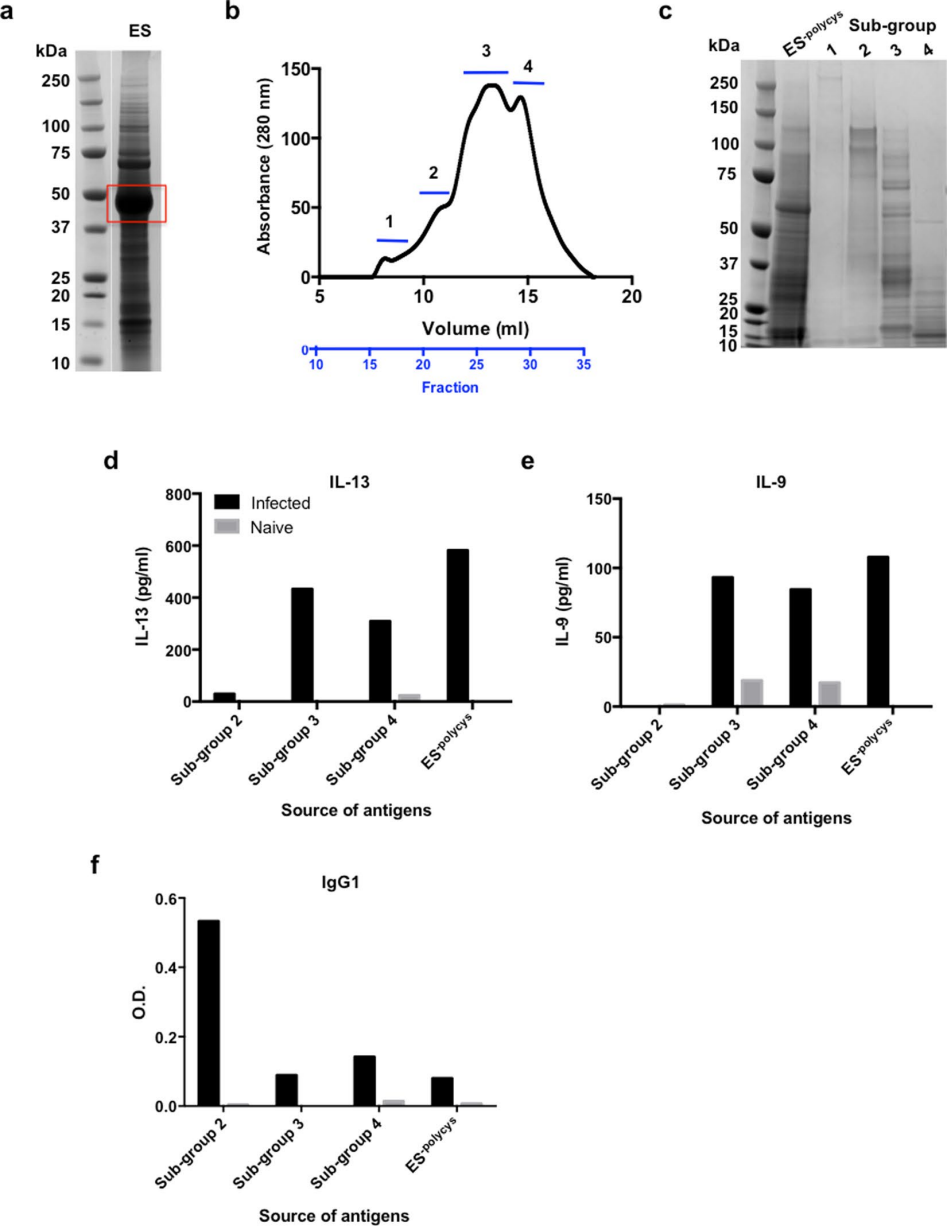
Division of ES^-polycys^ into four sub-groups and characterisation of their immunogenic properties. (**a**) Shows *T*. *muris* ES prior to removal of poly-cysteine and histidine tailed protein isoform 2 (indicated by red box). The two lanes shown in (**a**) are from different parts of the same gel. (**b**) Shows UV trace (absorbance at 280 nm) for ES^-polycys^ fractionation process, indicating which fractions were pooled to make sub-groups 1 to 4. (**c**) Shows SDS-PAGE separation of ES^-polycys^ and sub-groups 1 to 4. (**d**) and (**e**) show IL-13 and IL-9 production by lymphocytes in response to stimulation with sub-groups 2 to 4. Black bars indicate infection-primed lymphocytes taken from female C57BL/6 mice at day 20 p.i., while grey bars represent lymphocytes taken from age-matched female naïve C57BL/6 mice. (**f**) Shows anti-parasite antibody response (IgG1) for sub-groups 1 to 4. Black and grey indicate infected and naïve levels respectively. Left lanes in (**a**) and (**c**) show molecular weight markers in kDa.

The search for vaccine candidates was focussed on *T*. *muris* ES, since previous studies have shown that vaccination with this material stimulates protective immunity^18–20^. The most abundant component of adult ES is the poly-cysteine and histidine tailed protein isoform 2 (TMUE_s0083002300, indicated by red box in Fig. 1a)^12^, however on going work suggests that this protein is immunologically cryptic (Bancroft and Grencis, personal communication), and therefore it was removed from ES using nickel affinity beads, to enable focus on the other less abundant ES components (referred to here as ES^-polycys^).

Proteomic analysis revealed that *T*. *muris* ES^-polycys^ contains over 460 proteins (Table S1), and therefore it was necessary to develop methods to divide this material into smaller sub-groups in order to narrow down the search for protective immunogens. ES^-polycys^ was divided into four sub-groups using Superose 12 gel filtration chromatography (Fig. 1b and c). The amount of material in sub-group 1 was minimal, and therefore we have focussed on sub-groups 2 to 4. The cellular and humoral immune responses to antigen sub-groups 2 to 4 were investigated following a high dose *T*. *muris* infection, which primes the animal to make a protective Th2 response. Mesenteric lymph node (MLN) cell cultures from infected and naïve mice were re-stimulated with antigen sub-groups 2 to 4 to measure antigen specific cytokine production, and serum antibody levels (IgG1) specific for the different antigen sub-groups were also assessed. IL-13 and IL-9 cytokine production by infection-primed lymphocytes was highest in response to stimulation with sub-groups 3 and 4 (Fig. 1d and e), whereas the highest anti-parasite antibody levels were measured in response to sub-group 2 (Fig. 1f).

**Figure 2:**
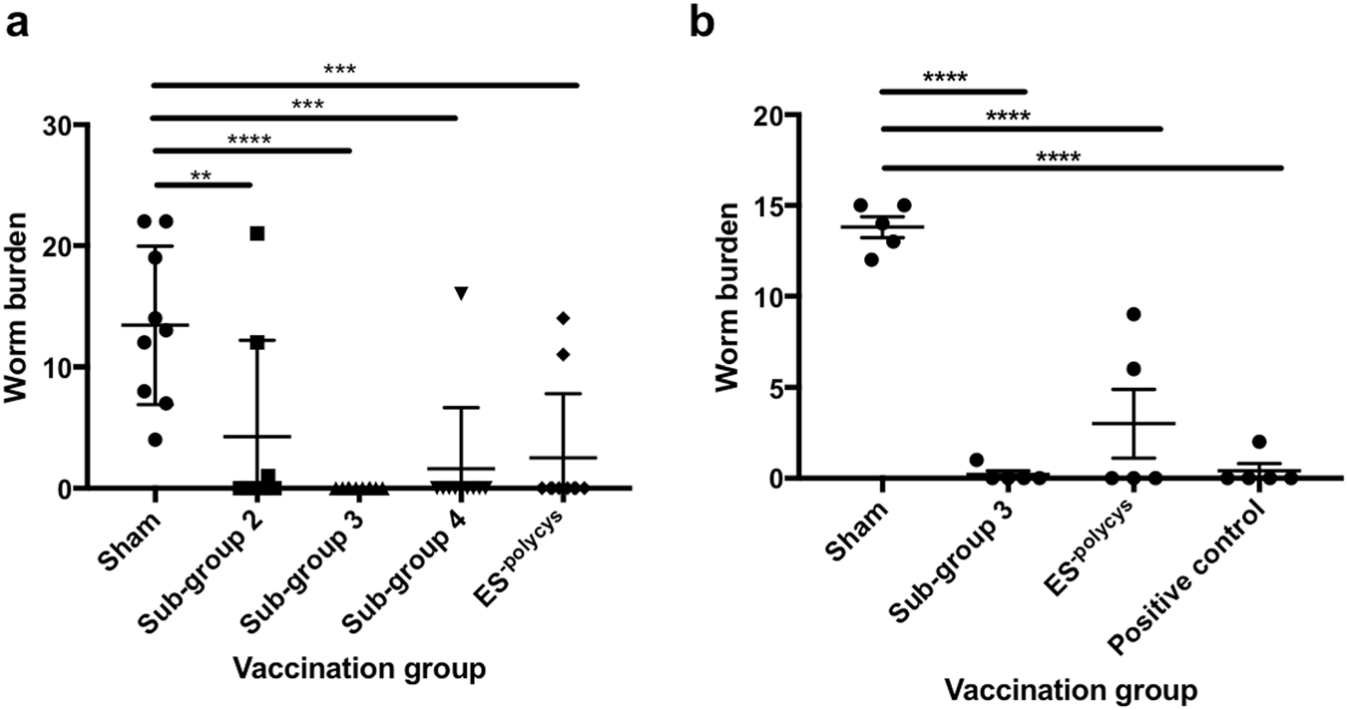
Vaccination with sub-group 3 or unfractionated ES^-polycys^ can induce long-lasting protective immunity. (**a**) Shows worm burden after s.c. vaccination of male C57BL/6 mice with 30 μg of sub-groups 2 to 4 followed by infection with 25 *T*. *muris* eggs 14 days later. Mice were vaccinated twice with the appropriate sub-group (14 days apart) and worm burdens were compared to those of the sham vaccination group. (**b**) Shows worm burden after s.c. vaccination of male C57BL/6 mice with 30 μg of sub-group 3 antigens or unfractionated ES^-polycys^ followed by infection with 25 *T*. *muris* eggs 50 days later. Sham indicates mice vaccinated with alum only, positive control indicates mice that were vaccinated twice with ES^-polycys^ (14 days apart) and then given a low dose infection 2 weeks after the second vaccination (as described in **a**). Error bars show SEM, central bar shows mean, ****P < 0.0001, ***P < 0.001, **P < 0.01.

Next, the protective properties of sub-groups 2 to 4 were investigated *in vivo*. Mice were vaccinated with each sub-group and then challenged with a low dose infection. Vaccination with all three sub-groups resulted in significantly lower worm burdens compared to the sham vaccination group, however vaccination with sub-group 3 consistently resulted in sterile immunity (Fig. 2a), and therefore this material formed the basis of the search for protective components going forward. These experiments also demonstrate that the protective properties of ES antigens can be correlated with Th2 cytokine production *in vitro*.

In order to determine whether vaccination with ES products induces long-lasting protection against a subsequent *T*. *muris* infection, i.e. a memory response, mice were vaccinated with either ES^-polycys^ or sub-group 3 and then challenged with 25 *T*. *muris* eggs a total of 50 days after the second vaccination. Vaccination with sub-group 3 antigens or unfractionated ES^-polycys^ led to a statistically significant reduction in worm burden by day 32 post challenge compared to the sham vaccination group (*P = *0.0001 for both, Fig. 2b). This was similar to the positive control group, which were vaccinated with ES^-polycys^ and infected with 25 *T*. *muris* eggs 14 days after the second vaccination, as described in Fig. 2a. These results suggest that vaccination with ES material can induce immunological memory.

**Figure 3:**
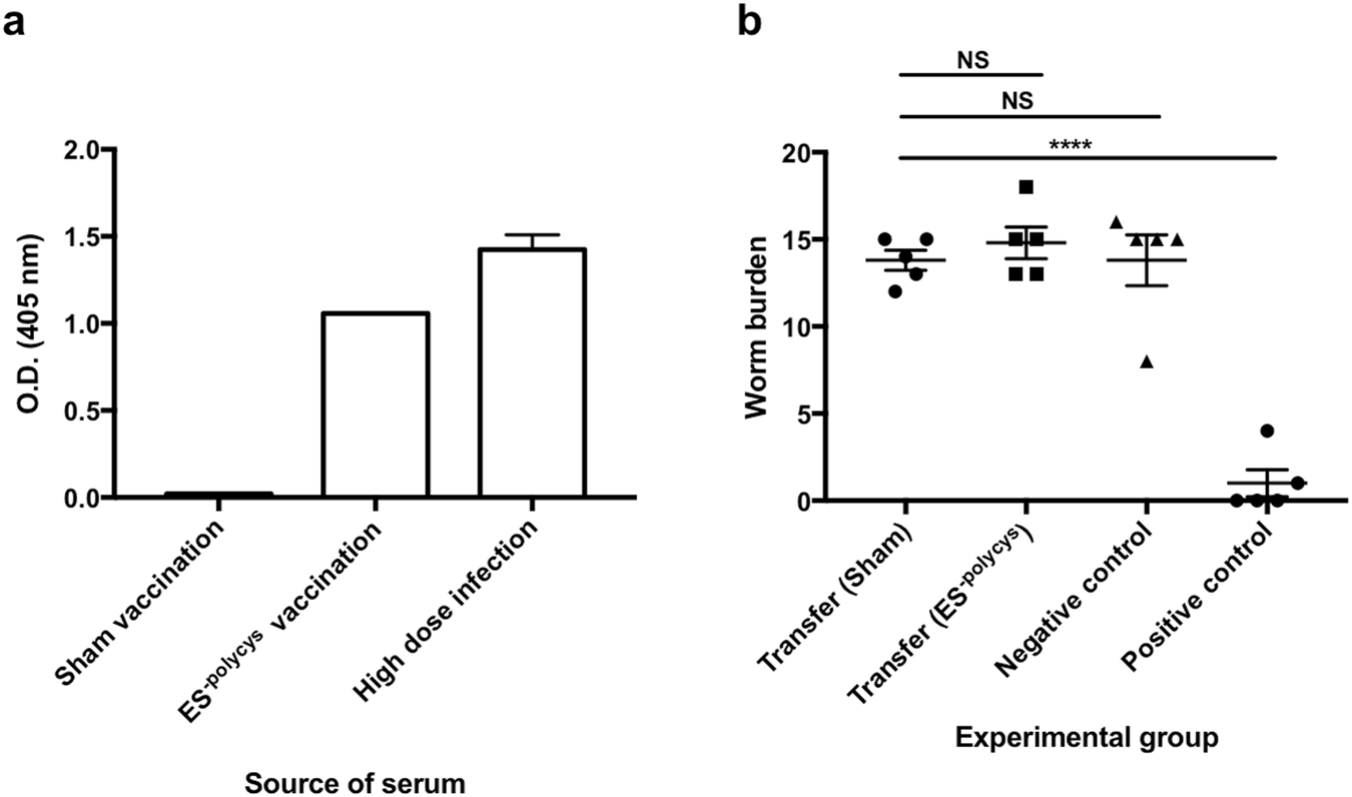
Transfer of serum from ES^-polycys^ vaccinated mice does not confer protection. (**a**) Shows parasite-specific IgG1 levels found in mice given a sham (alum only) vaccination, vaccinated with ES^-polycys^ or given a high dose infection with *T*. *muris*. (**b**) Shows worm burdens of mice following transfer of serum from sham (alum only) or ES^-polycys^ vaccinated mice, followed by low dose infection. The worm burden of the negative (sham vaccination followed by low dose infection) and positive control (ES^-polycys^ vaccination followed by low dose infection) groups are included for comparison. ****P < 0.0001, NS = non-significant. Error bars indicate SEM.

Many vaccines rely on the generation of protective antibodies to underpin their efficacy based upon the known importance of antibodies in naturally induced immunity to the infection concerned. Previous work has shown that acquired immunity to *T*. *muris* is mediated by IL-13, derived from CD4^+^ T cells^21–23^ with a minimal role for antibody^24,25^. Similar to infection with a high dose of *T*. *muris*, vaccination with ES^-polycys^ did generate significant levels of parasite specific IgG1 antibody (Fig. 3a), however passive transfer of serum from ES^-polycys^ vaccinated mice did not confer protection to naïve mice (Fig. 3b), suggesting that the role of antibody in vaccination induced protection against *T*. *muris* is minimal. We therefore employed a screening method based on identifying components within sub-fractions of ES^-polycys^ that induced antigen specific IL-13 and IL-9 production in order to identify immunogenic candidates.

### Identification of potential immunogenic candidates

Immunogenic candidates were identified using a combination of chromatography, mass spectrometry and *in vitro* measurements of T cell cytokine production. The approach involved two size exclusion chromatography steps – the first step was to fractionate ES^-polycys^ using Superose 12 gel filtration chromatography, while the second step involved further fractionating the material encompassing sub-group 3 (Fig. 1b) by Superdex 75 gel filtration chromatography.

**Figure 4:**
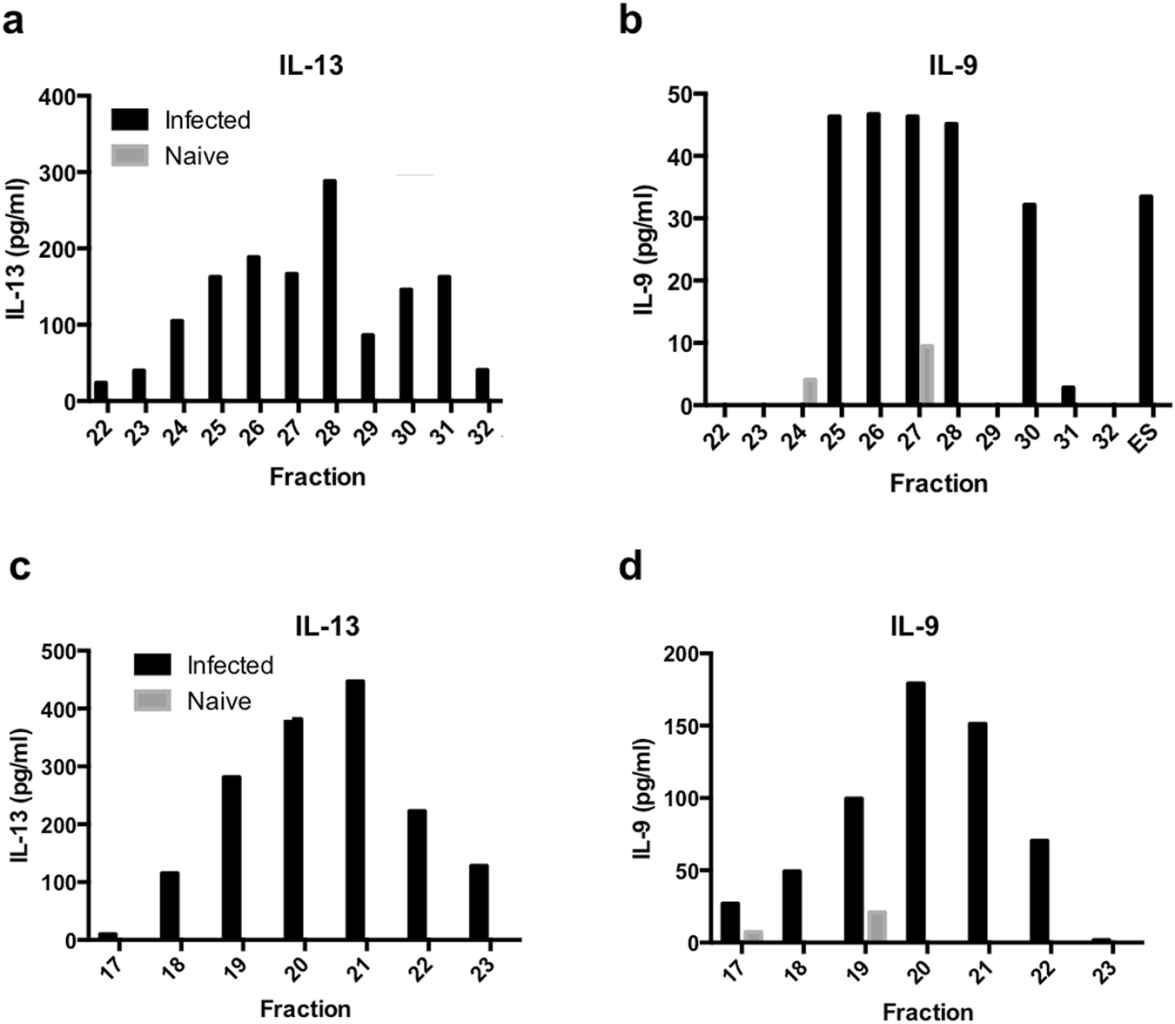
Identification of immunogenic candidates within Superose 12 and Superdex 75 fractions. ES^-polycys^ was fractionated using Superose 12 gel filtration media, however, instead of pooling the resulting fractions to make sub groups 1 to 4 as described in Fig. 1, fractions 22 to 32 were used to stimulate infection-primed lymphocytes from female C57BL/6 mice (day 20 p.i.) and lymphocytes from age-matched naïve female C57BL/6 mice. Supernatant IL-13 and IL-9 levels were measured after 42 hours and are displayed in (**a**) and (**b**). Superose 12 fractions 24 to 27 (corresponding to sub-group 3 in Fig. 1b & c) were fractionated using Superdex 75 gel filtration media. Fractions 17 to 23 from the Superdex 75 column were used to stimulate infection-primed lymphocytes from female C57BL/6 mice (day 20 p.i.) and lymphocytes from age-matched naïve C57BL/6 mice. Supernatant IL-13 and IL-9 levels were measured after 42 hours and are displayed in (**c**) and (**d**).

Infection-primed and naïve lymphocytes were stimulated with 25 μg of each of the Superose 12 fractions 22 to 32 (Fig. S1) and supernatant cytokine levels were measured after 42 hours by cytometric bead array. There were two major peaks of IL-13 production by infection-primed lymphocytes – the first was between fractions 24 and 27 and the second was between fractions 29 and 31 (Fig. 4a). IL-13 production was also high in response to fraction 28 – this is likely due to an overlap in the immunogenic material found in the two IL-13 peaks. IL-9 production by infection-primed lymphocytes was also highest in response to fractions 25 to 28, with a second peak at fraction 30 (Fig. 4b). There was very little IL-13 and IL-9 produced by naïve lymphocytes in response to stimulation with these fractions, suggesting that these fractions contain parasite-specific antigens that drive Th2 cytokine release during acute infection.

The protein content of fractions 22 to 32 was analysed by tandem mass spectrometry of tryptic peptides, which identified 325 proteins across the eleven fractions. For each fraction, a standardised amount of protein (10 μg) was used for mass spectrometry analysis to enable comparison of protein abundance between fractions, and total spectral count (the total number of spectra detected for each protein) was used as a surrogate for protein abundance^26^. Proteins whose spectral count peaked around fractions 24 to 27 were considered potential immunogenic candidates, as high levels of IL-13 were detected in these fractions (Fig. 4a), and vaccinating mice with these antigens in the form of sub-group 3 protected mice against a subsequent *T*. *muris* infection (Fig. 2a). The number of potential immunogenic candidates identified in this step was 62, and these are shown in Table S2.

The second size exclusion chromatography step involved fractionating the IL-13 inducing fractions (Superose 12 fractions 24 to 27 that corresponded to sub-group 3 in Fig. 1b & c) using Superdex 75 gel filtration media. Protein was eluted from the Superdex 75 column across seven fractions (Fig. S2). Infection-primed lymphocytes were re-stimulated with each of these fractions and supernatant cytokine production was measured by cytometric bead array.

The pattern of IL-13 production by infection-primed lymphocytes in response to the Superdex 75 fractions was very clear; there was a steady increase in IL-13 in response to stimulation with fractions 17 to 21. IL-13 production peaked at fraction 21, and this was followed by a decrease in IL-13 production in response to fractions 22 and 23 (Fig. 4c). The pattern of IL-9 production by infection-primed lymphocytes was very similar, although cytokine production peaked slightly earlier, at fraction 20 (Fig. 4d). Naïve lymphocytes produced very little IL-13 and IL-9 in response to stimulation with fractions 17 to 23, again demonstrating that these fractions contain parasite-specific antigens capable of driving Th2 cytokine production during acute infection.

The protein content of each fraction from the Superdex 75 column (fractions 17 to 23) was analysed by tandem mass spectrometry of tryptic peptides, and 190 proteins were identified across the seven fractions. For each fraction, a standardised amount of protein (10 μg) was used for mass spectrometry analysis to enable comparison of protein abundance between fractions, again using spectral count as a surrogate for protein abundance^26^. A total of 71 proteins were identified whose abundance peaked around fractions 20 to 22, matching the peak in IL-13 and IL-9 levels. These proteins are listed in Table S3.

**Figure 5:**
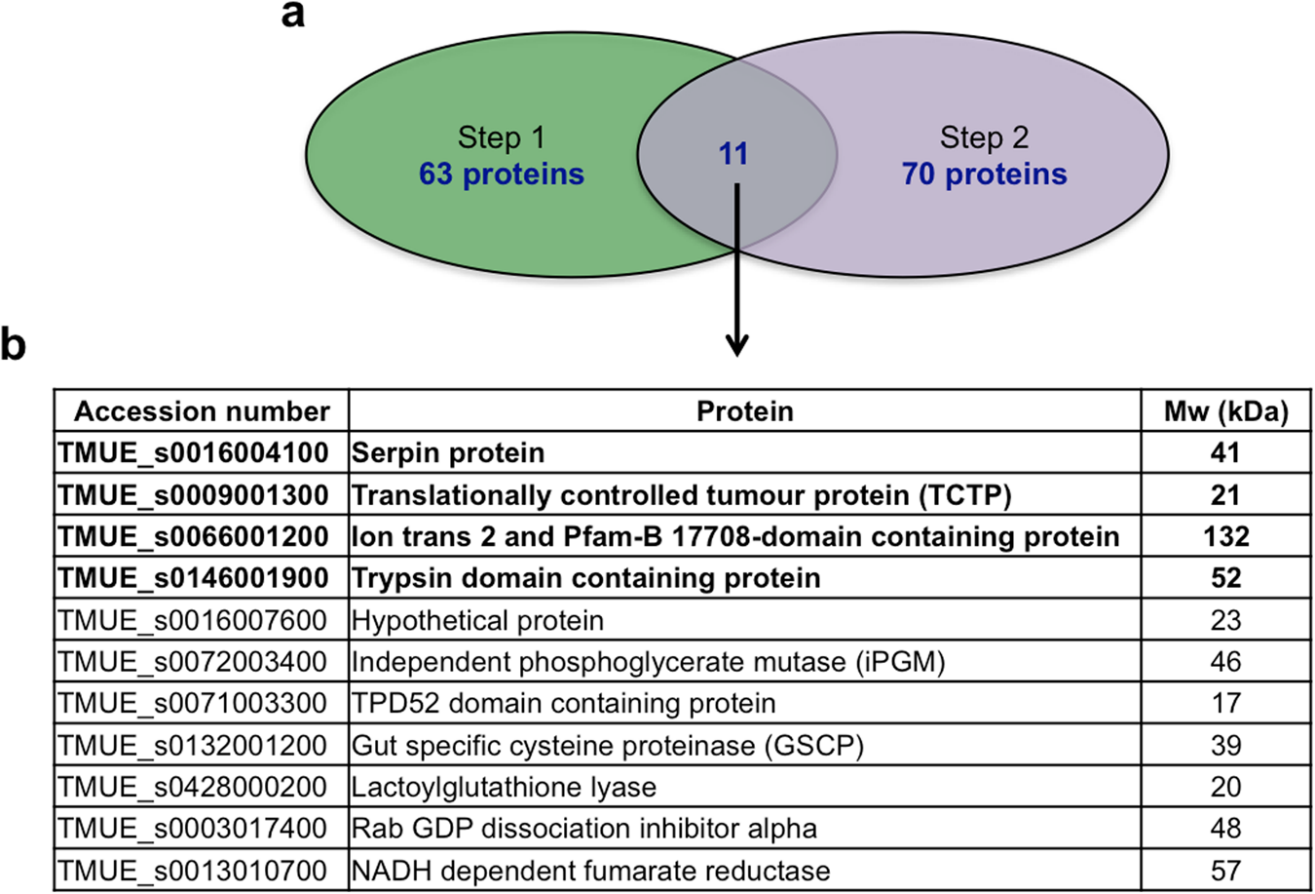
Strategy for selecting immunogenic candidates for further investigation. (**a**) Eleven immunogenic candidates were identified based on the overlap between the proteins identified from the Superose 12 (Step 1) and Superdex 75 (Step 2) fractionation steps. (**b**) List of potential immunogenic proteins. Mw = molecular weight in kDa.

Eleven potential immunogenic candidates were identified based on the overlap between the 62 candidates identified in the first size exclusion chromatography step (using Superose 12 gel filtration media) and the 71 candidates identified in the second step (using Superdex 75 gel filtration media, Fig. 5a). These proteins are listed in Fig. 5b and highlighted in bold in Tables S2 and S3.

## Discussion

Here, we show that vaccination of male C57BL/6 mice with ES^-polycys^ induces long-lasting protective immunity against *T*. *muris* infection. This is particularly noteworthy given that the mice were challenged with an infection dose that would ordinarily induce a Th1 dominated response, which would progress to chronicity, and this infection regime is more reflective of a natural infection^14^. These data suggest that ES antigens are able to prime the immune system to induce a Th2 response that mediates worm expulsion. It is generally accepted that in order to confirm that memory cells are generated in mouse studies, at least 30 days should have elapsed post antigen dosing^27–29^. Here, mice were infected a total of 50 days after the second vaccination, which strongly suggests that vaccination with *T*. *muris* ES^-polycys^ stimulates immunological memory.

Due to the large number (>460) of proteins identified within ES, it was deemed necessary to divide the ES^-polycys^ into smaller sub-groups in order to narrow down the search for protective antigens. ES^-polycys^ was divided into four sub-groups on the basis of hydrodynamic size, and the sub-groups that afforded the greatest protection following vaccination (sub-groups 3 and 4) also induced the greatest IL-13 (and IL-9) secretion by infection-primed MLN lymphocytes. This suggests that the protective properties of ES components can be predicted by assaying for antigen specific Th2 cytokine production.

We also showed that transfer of serum from ES^-polycys^ vaccinated mice did not protect naïve mice from chronic infection, suggesting that anti-parasite antibodies do not play a major role in vaccine induced worm expulsion. Based on previous studies showing that natural acquired immunity is driven by IL-13 (and to a lesser extent IL-9) production by CD4^+^ T cells^17,21–23^, we reasoned that a screening method involving the identification of IL-13 inducing *T*. *muris* antigens could identify potent vaccine candidates.

Immunogenic candidates were identified using a chromatography and mass spectrometry-based approach coupled to a measure of T cell cytokine production. This approach is supported by Santos and co-workers, who used ion exchange chromatography to divide *T*. *trichiura* homogenate into different sub-groups in order to identify immunomodulatory material^30^. Vaccine candidates for other gastrointestinal helminths have been identified using antibody-based screening methods, whereby immune sera (and other sources of protective antibodies) were used to probe for antigenic material^31–37^. However, such an approach may not be effective for *T*. *muris*, given that antibodies do not appear to play a major role in vaccine-induced resistance. Using our approach, eleven immunogenic candidates were identified. All of these had direct homologues in *T*. *trichiura*, which emphasises the potential for the *T*. *muris* model to inform vaccine design for *T*. *trichiura*. This dataset has highlighted a number of possibilities for vaccine candidates going forward, including iPGM, serpin and TCTP, since antigenic homologues of these proteins have been reported in vaccination studies against other parasites^38–56^, however we acknowledge that there may be additional vaccine candidates present in the chromatographic separations reported here that warrant further exploration.

The immunogenic potential of *Brugia malayi* iPGM has been highlighted by vaccination and RNA interference experiments^57,58^. Vaccination with recombinant iPGM from *B*. *malayi* protected BALB/c mice from a subsequent infection with a 58% reduction in worm burden, while RNA silencing of female adult worms resulted in a 90% decrease in worm motility and an 80% reduction in the number of microfilariae released^57,58^. Furthermore, only 55% of L3 larvae treated with iPGM-specific siRNA survived to adulthood, suggesting that iPGM plays an important role across several life cycle stages^58^.

GSCP may also make an effective vaccine candidate for *Trichuris* parasites, as it has been shown for other gastrointestinal nematodes that cysteine proteases are important for worm survival^40,41^. Treatment of *A*. *ceylanicum* infected hamsters orally with cysteine proteinase inhibitors reduced worm burdens by over 90%, and had potent larvicidal activity against eggs and first-stage larvae *in vitro*^40^. An older study showed that vaccinating dogs with a recombinant *A*. *caninum* cysteine protease induced IgG antibodies capable of binding to (and presumably neutralising) native cysteine proteases in the gut of worms^41^. Little is known about the feeding processes of *Trichuris* parasites^12^, however gut cysteine proteinases may be involved in the breakdown of nutrients by the worm, and therefore targeting these may reduce the viability of the parasite.

Vaccination of BALB/c mice with a recombinant *Trichinella spiralis* serpin led to a significant reduction in the number of adult worms resulting from a subsequent infection^42^. It is hypothesised that parasitic nematodes may have evolved serpins in order to block host serine proteases, for example hookworm serpins are responsible for the parasite’s anti-coagulation properties^43–45^. However, by interfering with host processes, serpins themselves may be targeted by the host immune system^46^. For example, *B*. *malayi Bm-*SPN-2 is targeted by T cells and antibodies in humans and mice^47^. Foth and colleagues identified 80 serpin proteins in *T*. *muris*, and found that the majority of these share similarities with secretory leukocyte peptidase inhibitors (SLPIs)^12^. Mammalian SLPIs often have immunomodulatory or anti-microbial properties in addition to their protease inhibitor activity^48–50^. Foth and colleagues reason that *Trichuris* serpins could perform similar roles, and therefore may be targeted by the host immune system as part of the evolutionary arms race between host and pathogen^12^.

TCTPs are expressed by a wide range of organisms, including yeast, protozoa, nematodes and mammals^51^. TCTPs are highly expressed in nematode eggs (both free living nematodes such as *Caenorhabditis elegans* and parasitic nematodes such as *Ostertagia ostertagi*, *Teladorsagia circumcincta* and *Haemonchus contortus*, where they appear to play an important role in egg production^51^. *B*. *malayi* TCTP has been shown to have antioxidant properties, while TCTPs isolated from *Trypanosoma brucei gambiense* have been shown to bind to and alter the growth pattern of bacteria in the mid gut of the tsetses fly, which is the vector for this parasite^52,53^. Taylor and colleagues showed that vaccination of BALB/c mice with recombinant *Plasmodium falciparum* TCTP prior to infection with a lethal *P*. *yoelli* strain reduced parasitaemia during the early stages of infection, suggesting that TCTP is required for an infection to establish^54^. Other studies show that *P*. *falciparum* and *S*. *mansoni* TCTP proteins are homologues of mammalian histamine-releasing factor and demonstrate that these proteins stimulate histamine release from basophils/mast cells and recruit eosinophils^55,56^.

In conclusion, the work presented here provides a framework for identifying immunogenic candidates using a chromatography/proteomics approach coupled to measurements of T cell cytokine release. A number of interesting observations were made relating to the characteristics of the immune response induced by vaccination with ES, including the length of the protection conferred by vaccination. Several promising immunogenic candidates were identified from these experiments, including serpin, TCTP, GSCP and iPMG. This provides a rational basis for pursuing the protective capacity of these candidates in vaccination studies going forward.

## Materials and Methods

### Maintenance of animals and parasites and ES^-polycys^ preparation

C57BL/6 mice (Envigo) were maintained in individually ventilated cages at 22 °C ± 1 °C and 65% humidity with a 12 h light-dark cycle. Mice had free access to food and water, and all procedures were carried out on mice 6–8 weeks of age or older, under the Home Office Scientific Procedures Act (1986). Animals were humanely killed by CO_2_ inhalation followed by terminal exsanguination.

All experimental protocols were approved and performed in accordance with the regulations of the Home Office Scientific Procedures Act (1986), Project licence 70/8127 and the University of Manchester Animal Welfare and Ethical Review Body (AWERB). The experiments conform to the ARRIVE guidelines. All methods were carried out in accordance with the above relevant guidelines and regulations.

Parasite maintenance and ES collection from adult worms were carried out as described previously^57^. The poly-cysteine and histidine tailed protein isoform 2 was removed from adult ES samples using Ni-NTA agarose beads (Qiagen).

### Mass spectrometry and proteomic analysis of ES^-polycys^ components

Tryptic digestion and mass spectrometry analysis of ES material was carried out as described previously^58^. The results were analysed using Mascot MS/MS ion search (Matrix Science) and searched against the *T*. *muris* proteome, version 2.1 (Sanger Centre). The Sanger Centre FTP site (ftp://ftp.sanger.ac.uk/pub/project/pathogens/Trichuris/muris/genes/) was used to search for *T*. *muris* protein sequences. Scaffold Proteome Software (Scaffold, USA) was used to calculate the exclusive unique peptide count for each protein (criteria set to 95% protein threshold, 50% peptide threshold, minimum 2 peptides identified).

### *In vitro* assessment of humoral and cellular response to ES^-polycys^ components during acute infection

Female C57BL/6 mice were infected with 200 *T*. *muris* eggs by oral gavage and sacrificed at day 20 p.i. The humoral and cellular response to ES antigens was compared to age-matched naïve controls. Serum IgG1 antibody response to ES material was measured by ELISA as described previously^57^. Cell culture and cytokine analysis were also performed as described previously^57^.

### Vaccination studies

Male C57BL/6 mice were used for all vaccination studies. These mice were vaccinated subcutaneously (s.c.) with 30 μg of ES material followed by a second vaccination 14 days later (50 days later for long-term vaccination study) with 15 μg antigen. Vaccinations were formulated with Imject alum (Thermo) in a 1:1 (v/v) ratio of antigen to adjuvant, and 100 μl was administered using a 25-gauge needle (BD Microlance). Sham vaccinations were performed with saline in 1:1 dilution with alum. Mice were infected with 25 *T*. *muris* eggs 28 days after the first vaccination and worm burdens were assessed at day 32 p.i.

### Serum transfer

Male C57BL/6 mice were vaccinated with ES^-polycys^ or sham vaccinated as described above. These mice were sacrificed 14 days after the second vaccination and serum was collected following cardiac puncture. Naïve male C57BL/6 mice were injected intraperitoneally with 500 μl of heat treated (54 °C for 30 minutes) serum from ES^-polycys^ or sham-vaccinated mice. These mice were infected with 25 *T*. *muris* eggs by oral gavage immediately following the serum transfer and were sacrificed at day 32 p.i. to assess worm burdens.

### Fractionation of adult ES^-polycys^ by gel filtration chromatography

ES^-polycys^ was prepared in 25 mM Tris, 10 mM NaCl (pH 7.4), and fractionated using Superose 12 gel filtration media under the control of an ÄKTAprime plus purification system (all GE Healthcare). Sub-group 3 antigens (resulting from the pooling of Superdex 75 fractions 24 to 27) were further fractionated using Superdex 75 gel filtration media. For both fractionation steps, the column flow rate was set to 0.5 ml/min and protein was eluted in 0.5 ml fractions with 25 mM Tris, 10 mM NaCl (pH 7.4). The column effluent was monitored for UV absorbance at 280 nm. Unicorn software was used to control the fractionation process, and elution profiles were viewed using the PrimeView software (GE Life Sciences). The protein content of fractions was monitored by SDS-PAGE as described previously^59^.

### Assessing protein concentration of samples

Protein concentration was assessed using a bicinchoninic acid assay kit (Fisher Scientific), according to the manufacturer’s instructions.

### Statistical analysis

All statistical analysis was carried out using GraphPad Prism 7. One-way ANOVA (followed by Dunn’s multiple comparison test) was performed for each experiment. Unless otherwise stated, error bars represent SEM. Statistical significance is represented by *(*P* < 0.05), **(*P* < 0.01), ***(*P* < 0.001) or ****(*P* < 0.0001).

### Data availability statement

All data generated or analysed during this study are included in this article (and its Supplementary Information files).

## Electronic supplementary material

Supplementary materials
