# Supplementary material for: Vaccination Against Whipworm: Identification of Potential Immunogenic Proteins in Trichuris muris Excretory/Secretory Material
Source: Sci Rep. 2018 Mar 14;8:4508. doi: 10.1038/s41598-018-22783-y (PMC5851985; doi:10.1038/s41598-018-22783-y)
Supplement: Supplementary file 1 — Supplementary materials [file 41598_2018_22783_MOESM1_ESM.pdf]

SUPPLEMENTARY MATERIALS

Vaccination Against Whipworm: Identification of Potential Immunogenic Proteins in *Trichuris muris* Excretory/Secretory Material

Rebecca K. Shears, Allison J. Bancroft, Catherine Sharpe, Richard K. Grencis* and David J. Thornton*.

Wellcome Trust Centre for Cell-Matrix Research and Manchester Immunology Group, School of Biological Sciences, Faculty of Biology, Medicine and Health, Manchester Academic Health Sciences Centre, University of Manchester, M13 9PT

*Co-corresponding authors

David Thornton, AV Hill Building, Faculty of Biology, Medicine and Health, University of Manchester, Oxford Road, Manchester, M13 9PT
E-mail: dave.thornton@manchester.ac.uk

Richard Grencis, AV Hill Building, Faculty of Biology, Medicine and Health, University of Manchester, Oxford Road, Manchester, M13 9PT
E-mail: richard.grencis@manchester.ac.uk

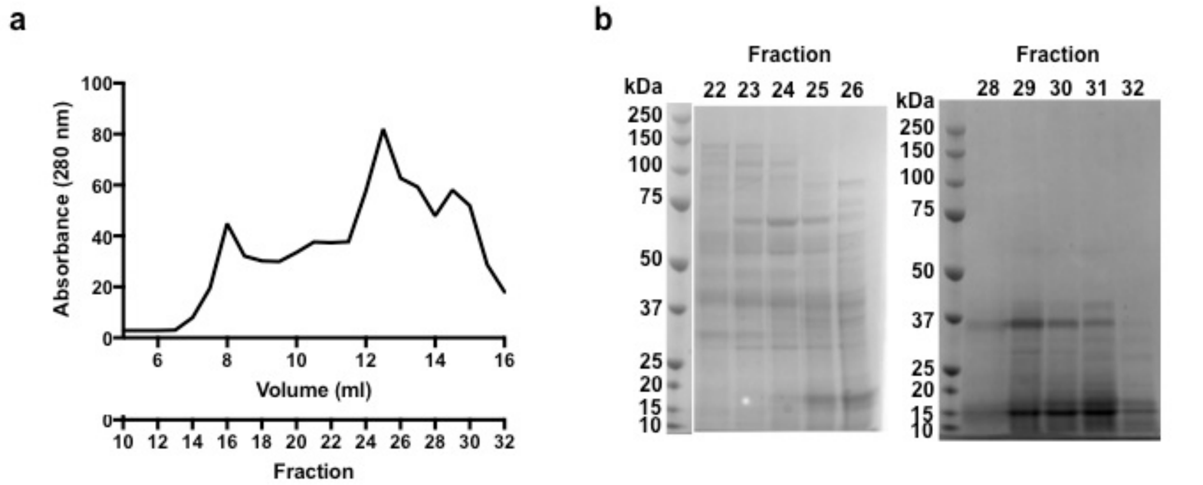

Figure S1. Fractionation of ES using Superdex 75 chromatography media. (a) Shows UV trace (absorbance at 280 nm) across the chromatographic separation, while (b) shows SDS-PAGE analysis of fractions 22 to 32 (left lane shows molecular weight markers in kDa).

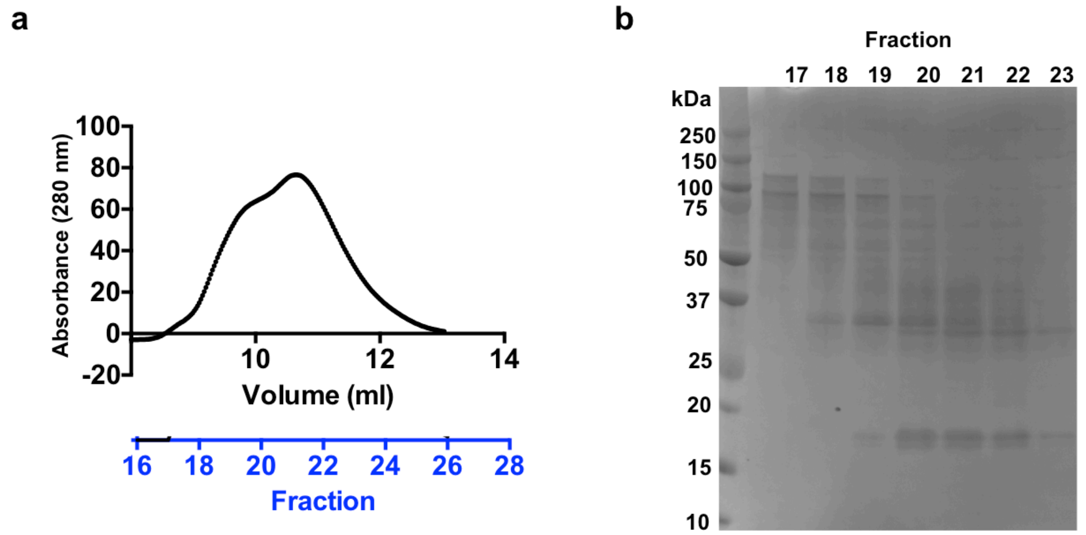

Figure S2. Fractionation of sub-group 3 antigens using Superdex 75 chromatography media. (a) Shows UV trace (absorbance at 280 nm) across the chromatographic separation, while (b) shows SDS-PAGE analysis of fractions 17 to 23 (left lane shows molecular weight markers in kDa).

Table S1

	Accession number	Protein	Mw (kDa)	Fraction																
				16	17	18	19	20	21	22	23	24	25	26	27	28	29	30	31	32
1	TMUE_s0327000100	Pfam-B 9093-domain containing protein	39	0	0	0	0	0	0	0	35	68	73	48	24	11	7	7	8	0
2	TMUE_s0027006900	myoglobin	18	6	6	0	3	5	0	2	0	2	0	4	5	19	31	41	18	19
3	TMUE_s0165000300	WAP type 'four disulfide core'	48	0	0	0	0	0	0	0	44	33	29	23	12	11	9	16	12	12
4	TMUE_s0201000900	Pfam-B 9093-domain containing protein	39	0	0	0	0	0	0	0	42	61	36	14	8	5	0	5	3	5
5	TMUE_s0103004200	epididymal secretory protein E1	35	0	0	0	0	0	0	0	0	2	0	0	0	13	22	48	25	20
6	TMUE_s0102000900	enolase	48	25	19	14	9	9	5	6	0	6	14	14	16	10	2	7	6	6
7	TMUE_s0029005700	actin	42	11	11	13	13	5	4	4	5	13	19	18	18	11	3	4	6	8
8	TMUE_s0086000700	78 kDa glucose regulated protein	73	0	0	0	0	0	0	0	11	11	10	12	5	0	2	3	5	3
9	TMUE_s0012009900	hypothetical protein	45	0	8	34	21	36	3	10	12	9	6	9	7	0	0	0	0	0
10	TMUE_s0003006100	hypothetical protein	225	7	5	4	7	6	3	7	6	11	8	9	10	7	5	5	7	3
11	TMUE_s0016011400	MULE and Pfam-B 516 and WAP-domain containing	71	0	0	0	0	0	0	0	2	10	15	20	29	16	12	12	9	11
12	TMUE_s0003007600	WAP type 'four disulfide core'	41	0	0	0	0	0	0	0	10	22	16	18	30	23	12	13	7	8
13	TMUE_s0003007100	hypothetical protein	162	5	5	10	15	21	7	4	6	11	6	9	8	8	6	3	4	2
14	TMUE_s0256000200	hypothetical protein	70	20	13	13	10	9	0	5	3	4	7	13	12	12	8	6	5	2
15	TMUE_s0250000100	hypothetical protein	28	4	0	4	2	3	2	4	15	26	20	17	14	11	4	11	7	7
16	TMUE_s0252000100	hypothetical protein	23	4	8	13	7	9	9	14	13	13	13	10	16	10	8	7	8	6
17	TMUE_s0091002200	Fasciclin-domain containing protein	81	17	15	13	16	32	14	18	0	0	0	0	0	0	0	0	0	0
18	TMUE_s0005007100	protein mig c; protein mig b; protein mig a	234	27	25	23	27	20	9	4	0	0	0	0	0	0	0	0	0	0
19	TMUE_s0009012900	hypothetical protein	52	19	20	24	19	12	7	12	5	4	2	0	0	0	4	0	0	0
20	TMUE_s0033006400	CAP-domain containing protein	35	3	2	3	2	11	54	53	0	0	0	0	0	0	0	0	0	0
21	TMUE_s0081001900	protein disulfide isomerase	55	0	0	0	0	0	0	0	18	33	25	24	10	9	6	0	4	2
22	TMUE_s0003006600	Pfam-B 13663-domain containing protein	164	6	5	5	10	8	0	2	15	9	3	4	0	3	5	8	6	5
23	TMUE_s0034001200	conserved hypothetical protein	43	4	3	2	6	6	9	26	17	12	5	6	4	3	2	4	2	4
24	TMUE_s0004002000	chymotrypsin inhibitor	16	0	0	0	0	0	0	0	0	0	0	2	18	39	14	13	8	4
25	TMUE_s0049005700	protein crumbs	382	8	10	16	20	25	12	10	0	0	0	0	0	0	0	0	0	0
26	TMUE_s0246000600	conserved hypothetical protein	41	0	0	0	0	0	3	19	20	25	16	18	10	7	0	0	0	0
27	TMUE_s0014002400	conserved hypothetical protein	39	8	0	4	0	2	0	5	7	7	7	8	8	8	4	11	6	8
28	TMUE_s0175000100	Pfam-B 9093-domain containing protein	34	25	12	19	10	15	7	3	0	0	0	0	0	0	0	0	0	0
29	TMUE_s0245000500	VWD and Vitellogenin N and DUF1943-domain conta	202	12	13	13	11	15	8	19	0	0	0	0	0	0	0	0	0	0
30	TMUE_s0191000800	Trypsin-domain containing protein	79	0	0	0	0	0	0	0	26	27	9	8	9	7	6	6	5	4
31	TMUE_s0030008500	conserved hypothetical protein	36	8	9	5	5	4	3	5	13	16	7	5	5	2	3	4	3	0
32	TMUE_s0028000200	Phosphoenolpyruvate carboxykinase GTP	80	0	0	0	0	0	0	0	12	19	14	16	13	9	2	4	0	5
33	TMUE_s0256000600	conserved hypothetical protein	48	18	16	12	8	9	3	5	0	0	3	7	9	9	0	9	6	0
34	TMUE_s0252000300	hypothetical protein	64	0	0	0	0	0	0	0	5	18	25	17	14	9	0	7	3	2
35	TMUE_s0081002200	protein L isoaspartate(D aspartate)	51	0	0	0	0	0	0	0	0	0	0	3	6	10	13	24	16	13
36	TMUE_s0111003300	protein disulfide isomerase A3	65	0	0	0	0	0	0	0	8	16	15	14	9	6	0	9	2	4
37	TMUE_s0083002300	poly cysteine and histidine tailed protein	43	0	0	0	0	0	0	0	6	2	6	4	7	6	3	13	7	12
38	TMUE_s0002015700	Vitellogenin N and VWD and C8 and DUF1943-doma	354	19	6	19	16	13	2	0	0	0	0	0	0	0	0	0	0	0
39	TMUE_s0177000800	heat shock protein 70	71	0	0	0	0	0	0	0	11	9	14	11	7	6	3	4	3	3
40	TMUE_s0037003800	NADP dependent malic enzyme, mitochondrial	71	24	17	10	5	4	4	20	0	0	0	0	0	0	0	0	0	0
41	TMUE_s0095005600	DUF290-domain containing protein	13	0	0	0	0	0	0	0	0	0	0	0	0	16	5	26	19	9
42	TMUE_s0016004100	Serpin protein	177	13	12	12	11	9	4	5	0	0	0	0	0	0	0	0	0	0
43	TMUE_s0033001500	CAP-domain containing protein	40	0	0	0	0	0	0	0	14	24	15	5	9	8	5	4	0	2
44	TMUE_s0023000300	Pfam-B 9093-domain containing protein	32	4	2	3	4	3	2	5	9	16	13	13	3	3	0	0	0	2
45	TMUE_s0003007400	peptidase, S1A subfamily	49	0	0	0	0	0	0	0	4	15	13	14	13	9	4	8	4	2
46	TMUE_s0010011900	E3 ubiquitin protein ligase TRIM9	86	0	0	0	11	0	0	0	0	0	0	0	0	0	0	0	0	0
47	TMUE_s0123000300	protein ketn d; protein ketn c; protein ketn b; protein ke	1721	0	0	0	0	0	0	0	3	3	2	2	4	4	8	2	3	0
48	TMUE_s0040000600	5' nucleotidase	66	10	6	8	8	12	11	22	0	0	0	0	0	0	0	0	0	0
49	TMUE_s0023000400	Pfam-B 9093-domain containing protein	42	0	0	0	0	0	0	0	0	3	9	14	14	8	6	8	6	4
50	TMUE_s0208001600	pathogeneis protein 1B	43	26	12	13	8	5	7	9	0	0	0	0	0	0	0	0	0	0
51	TMUE_s0015001100	pancreatic alpha amylase	66	13	10	14	11	12	4	10	0	0	0	0	0	0	0	0	0	0
52	TMUE_s0004006800	Trypsin-domain containing protein	129	0	0	0	0	0	0	0	4	5	6	3	4	4	0	8	3	4
53	TMUE_s0302000300	fructose bisphosphate aldolase class I	44	0	0	0	0	0	0	0	28	17	10	11	7	3	0	0	0	0
54	TMUE_s0129000500	hypothetical protein	21	3	2	3	0	0	0	0	0	0	0	0	0	12	6	11	13	5
55	TMUE_s0007001400	phosphoglycerate kinase	45	19	14	11	8	9	2	0	0	0	0	0	0	0	0	0	0	0
56	TMUE_s0003000700	histone H4	11	5	6	0	11	15	6	4	0	0	0	0	0	0	0	0	0	0
57	TMUE_s0175001500	porin	54	0	0	0	0	0	5	0	20	29	17	10	15	0	6	7	6	10
58	TMUE_s0006008700	thioredoxin	22	5	5	0	5	2	4	5	0	0	0	2	4	8	3	4	2	2
59	TMUE_s0033006300	conserved hypothetical protein	40	3	0	0	3	6	18	13	9	8	0	0	0	0	0	0	0	0
60	TMUE_s0138000200	A macroglobulin complement component family	165	6	4	4	6	10	8	14	0	0	0	0	0	0	0	0	0	0
61	TMUE_s0118000700	Trypsin-domain containing protein	33	0	0	0	0	0	0	0	0	0	0	0	0	2	2	20	11	8
62	TMUE_s0111003000	calreticulin	46	2	3	5	0	4	4	4	12	12	8	3	0	0	0	0	0	0
63	TMUE_s0008001600	DNase II-domain containing protein	42	0	0	0	0	0	0	0	8	4	6	0	0	5	0	6	4	5
64	TMUE_s0030007700	DNase II-domain containing protein	31	0	0	0	0	0	0	0	11	14	13	6	7	5	0	2	0	0
65	TMUE_s0019005500	tropomyosin	33	0	0	5	8	7	0	4	2	0	3	0	3	3	0	4	3	5
66	TMUE_s0031002800	hypothetical protein	17	0	0	0	0	0	0	0	0	3	5	6	9	12	3	6	4	3
67	TMUE_s0005004200	malate dehydrogenase	39	0	0	0	0	0	0	0	0	3	5	17	9	3	0	4	0	0
68	TMUE_s0187000500	glucose 6 phosphate isomerase	74	0	0	0	0	0	0	0	0	14	14	10	5	3	0	0	0	0
69	TMUE_s0269000200	triosephosphate isomerase	27	0	0	0	0	0	0	0	4	5	3	6	9	9	9	4	3	2
70	TMUE_s0009007500	14-3-3 protein	28	0	0	0	0	0	0	0	21	12	11	8	7	0	2	0	0	0
71	TMUE_s0058002100	conserved hypothetical protein	26	0	0	0	0	0	0	0	0	0	4	9	7	6	0	4	3	3
72	TMUE_s0094001000	glyceraldehyde 3 phosphate dehydrogenase	41	7	12	10	10	7	0	0	0	0	0	0	0	0	0	0	0	0
73	TMUE_s0049001600	Trypsin-domain containing protein	215	10	5	11	4	9	0	0	0	0	0	0	0	0	0	0	0	0
74	TMUE_s0004017900	WAP-domain containing protein	38	0	0	0	0	0	0	0	7	4	7	6	8	8	0	6	6	12
75	TMUE_s0066001200	Ion trans 2 and Pfam-B 17708-domain containing pro	132	0	0	0	0	0	0	0	0	0	3	6	3	6	3	5	3	5
76	TMUE_s0014013200	heat shock protein 90	81	0	0	0	0	0	0	0	9	9	8	5	3	4	3	0	0	0
77	TMUE_s0049001500	Trypsin-domain containing protein	139	10	8	8	8	6	2	2	0	0	0	0	0	0	0	0	0	0
78	TMUE_s0164001100	Neur chan LBD-domain containing protein	26	0	0	0	11	26	7	4	0	0	0	0	0	0	0	0	0	0
79	TMUE_s0082001700	calreticulin	47	3	6	0	0	2	4	9	12	9	6	5	0	0	0	0	0	0
80	TMUE_s0037003400	pathogeneis protein 1B	41	0	0	0	0	0	0	0	4	6	3	3	4	8	5	9	5	6
81	TMUE_s0033003400	Trypsin-domain containing protein	37	0	0	0	0	0	0	0	0	0	0	3	13	12	3	8	4	2
82	TMUE_s0092004500	Ldl recept a and Trypsin-domain containing protein	139	0	0	8	15	15	7	3	0	0	0	0	0	0	0	0	0	0
83	TMUE_s0232000600	conserved hypothetical protein	42	4	5	2	3	2	0	2	0	0	0	0	0	4	0	8	3	4
84	TMUE_s0048003500	serine protease	45	0	0	0	0	0	0	0	0	4	7	7	7	7	0	7	3	3
85	TMUE_s0038006700	32 kDa beta galactoside binding lectin	39	0	0	0	0	0	0	0	0	0	0	0	6	13	7	8	8	5
86	TMUE_s0193000500	HyTSR1 protein	64	5	4	8	10	10	3	5	0	0	0	0	0	0	0	0	0	0
87	TMUE_s0088003400	hypothetical protein	14	0	0	0	0	0	0	2	0	0	3	7	8	5	4	0	2	2
88	TMUE_s0208000600	WAP-domain containing protein	25	0	0	0	0	0	0	0	2	2	0	3	9	8	4	6	3	6
89	TMUE_s0137001000	hypothetical protein	43	5	7	0	0	0	0	0	0	0	0	6	11	6	2	0	0	0
90	TMUE_s0003006400	chymotrypsinogen B	161	0	0	0	0	0	0	0	12	7	3	0	0	0	3	3	0	0
91	TMUE_s0003006900	hypothetical protein	80	0	0	0	0	0	0	0	3	9	8	8	7	4	5	2	0	0
92	TMUE_s0085003400	Motile Sperm-domain containing protein	20	0	0	0	0	0	0	0	2	2	2	5	11	3	3	3	0	0
93	TMUE_s0069002100	hypothetical protein	99	0	0	0	0	0	0	0	0	0	0	3	3	0	0	8	3	7
94	TMUE_s0002013500	conserved hypothetical protein	56	5	3	3	4	4	0	0	0	0	3	4	3	0	0	0	2	0
95	TMUE_s0284000300	hypothetical protein	45	0	0	0	0	0	0	0	3	0	0	0	3	0	0	0	0	0
96	TMUE_s0099000600	cytosol aminopeptidase	52	7	8	2	4	7	4	11	0	0	0	0	0	0	0	0	0	0
97	TMUE_s0010008300	onchocystatin	17	0	0	3	0	0	2	3	2	2	3	0	3	3	0	3	4	9
98	TMUE_s0001022900	conserved hypothetical protein	40	5	0	0	3	5	4	4	6	6	2	0	0	0	0	0	0	3
99	TMUE_s0084003500	CAP-domain containing protein	39	0	0	0	0	0	0	0	3	6	9	10	7	6	0	3	0	2
100	TMUE_s0093000600	protein disulfide isomerase A6	48	0	0	0	0	0	0	0	0	5	6	8	3	5	2	4	4	2
101	TMUE_s0134003400	protein sma d; protein sma c; protein sma b	469	0	0	0	0	0	0	0	0	3	2	0	0	2	0	0	0	0
102	TMUE_s0116000200	hypothetical protein	21	0	0	0	0	0	0	0	0	0	2	4	4	5	2	5	6	5
103	TMUE_s0077002200	hypothetical protein	10	0	0	0	0	0	0	0	0	0	2	2	6	5	3	7	4	4
104	TMUE_s0042004400	Low density lipoprotein receptor repeat	164	0	0	0	0	0	0	0	3	2	5	4	5	3	4	3	0	2
105	TMUE_s0015011200	protein unc g; protein unc f; protein unc d; protein unc	761	0	0	0	0	0	0	0	0	3	3	4	0	5	4	3	0	2
106	TMUE_s0028001200	VAB 10A protein	829	0	0	0	0	0	0	0	3	0	0	0	6	0	4	0	0	0
107	TMUE_s0071005500	angiotensin converting enzyme	153	9	10	4	4	5	0	0	0	0	0	0	0	0	0	0	0	0
108	TMUE_s0038003700	HEAT and Pfam-B 3355-domain containing protein	287	3	0	0	0	0	0	0	0	2	4	2	2	2	2	0	0	0
109	TMUE_s0078002600	His Phos 2-domain containing protein	43	0	0	0	0	0	0	0	9	15	7	3	5	4	0	0	0	0
110	TMUE_s0032002300	Muscle M line assembly protein unc 89	740	0	5	0	3	7	2	0	0	0	0	0	0	0	0	0	0	0
111	TMUE_s0019006300	hypothetical protein	39	11	4	5	10	3	2	2	0	2	0	0	0	0	0	0	0	0
112	TMUE_s0093001800	vacuolar protein sorting associated protein 52	175	0	0	0	0	0	0	0	2	5	0	4	4	2	3	2	0	0
113	TMUE_s0035005100	Peptidase A17 and rve-domain containing protein	127	0	0	0	0	0	0	0	0	0	0	0	6	0	4	0	0	0
114	TMUE_s0165000700	cysteine glycine protein 2	12	0	0	0	0	0	0	0	0	0	3	5	4	3	0	5	5	5
115	TMUE_s0076003900	trans sialidase	156	0	0	0	0	0	0	0	3	2	2	6	3	3	0	3	4	4
116	TMUE_s0048003100	vinculin	123	0	0	0	3	0	0	0	0	2	2	3	0	0	0	0	0	0
117	TMUE_s0005011400	peptidyl prolyl cis trans isomerase 7	23	0	0	0	0	0	0	0	0	0	0	0	2	0	0	7	5	16
118	TMUE_s0053002000	proteasome subunit alpha type 5	26	7	4	9	11	4	2	0	0	0	0	0	0	0	0	0	0	0
119	TMUE_s0034001100	LRR 8 and PP2C and LRR 4 and LRR 1-domain cont	170	0	0	0	0	0	0	0	10	14	5	3	0	0	0	0	0	0
120	TMUE_s0003012100	peroxidasin	156	3	2	0	2	3	0	0	0	0	0	2	0	0	0	0	2	0
121	TMUE_s0098002000	EF hand family protein	35	0	0	0	0	0	0	0	0	3	3	3	2	5	2	6	3	4
122	TMUE_s0101002800	CBM 14-domain containing protein	96	0	2	2	7	12	12	6	0	0	0	0	0	0	0	0	0	0
123	TMUE_s0013010700	NADH dependent fumarate reductase	57	0	0	0	0	0	0	0	0	0	8	11	7	7	0	0	0	0
124	TMUE_s0232000400	Pro isomerase-domain containing protein	21	0	0	0	0	0	0	0	0	0	0	0	0	0	0	6	12	16
125	TMUE_s0042001800	Transmembrane cell adhesion receptor mua 3	276	0	0	0	6	14	6	0	0	0	0	0	0	0	0	0	0	0
126	TMUE_s0036003800	parathyroid hormone 2 receptor	45	2	0	0	0	0	0	0	2	6	5	7	6	4	0	3	0	0
127	TMUE_s0371000200	Pfam-B 9093-domain containing protein	37	15	13	14	8	10	5	0	0	0	0	0	0	0	0	0	0	0
128	TMUE_s0097002200	Dihydrolipoyl dehydrogenase	53	0	0	0	0	0	0	0	0	12	5	8	0	2	0	0	0	0
129	TMUE_s0047003900	CAP-domain containing protein	58	0	0	0	0	0	0	0	0	0	2	2	0	4	3	6	7	5
130	TMUE_s0085003200	cysteine rich protease inhibitor	39	0	0	0	0	0	0	0	0	0	2	5	2	4	0	3	3	8
131	TMUE_s0008001500	deoxyribonuclease II family protein	32	8	4	4	5	3	4	5	0	0	0	0	0	0	0	0	0	0
132	TMUE_s0119001000	membrane metallo endopeptidase 1 like	173	5	3	5	4	8	2	0	0	0	0	0	0	0	0	0	0	0
133	TMUE_s0024005900	DUF1759 and rve and DnaJ and DnaJ CXXCXGXG a	307	0	0	0	0	0	0	0	0	2	2	0	3	0	4	0	0	0
134	TMUE_s0005013800	puromycin sensitive aminopeptidase	97	11	6	7	6	6	0	4	0	0	0	0	0	0	0	0	0	0
135	TMUE_s0028001200	VAB 10A protein	829	0	3	2	0	4	0	0	0	0	0	0	0	0	0	0	0	0
136	TMUE_s0281000600	CBM 14 and TIL-domain containing protein	202	0	0	0	0	0	0	0	0	2	0	5	7	6	0	2	3	2
137	TMUE_s0201000800	conserved hypothetical protein	51	4	4	0	2	3	0	0	2	5	3	0	0	0	0	0	0	0
138	TMUE_s0074002600	Alcohol dehydrogenase NADP+ A	75	0	0	0	0	0	0	0	0	0	0	0	8	10	2	3	3	0
139	TMUE_s0078004700	copper:zinc superoxide dismutase	20	0	0	0	0	0	0	0	0	0	0	4	11	8	4	7	0	3
140	TMUE_s0001020000	DnaJ and DUF290-domain containing protein	39	0	0	0	0	0	0	0	0	0	0	0	0	9	4	6	2	2
141	TMUE_s0095003100	Integrin beta and EGF 2-domain containing protein	51	0	0	0	0	0	0	0	9	10	7	7	4	0	0	0	0	0
142	TMUE_s0149002500	DDE Tnp IS1595 and rve and Pfam-B 9093-domain c	112	0	0	0	0	0	0	0	0	0	0	4	4	0	0	2	0	0
143	TMUE_s0012007100	Pfam-B 19232 and CAP-domain containing protein	58	0	0	0	0	0	0	0	5	7	7	6	3	2	0	0	0	0
144	TMUE_s0059000800	hypothetical protein	20	0	0	0	0	0	0	0	0	0	0	0	0	2	3	10	7	8
145	TMUE_s0134000500	CAP-domain containing protein	32	0	0	0	0	0	0	0	5	6	6	5	5	2	0	2	0	0
146	TMUE_s0011008900	myoglobin	17	2	2	0	0	0	0	0	0	0	0	0	0	4	9	7	4	3
147	TMUE_s0005010900	neurogenic locus notch protein	367	2	4	4	7	3	0	0	0	0	0	0	0	0	0	0	0	0
148	TMUE_s0066002300	hypothetical protein	16	0	0	0	0	0	0	0	0	5	4	3	5	5	0	4	3	3
149	TMUE_s0055003300	elongation factor 2	91	0	0	0	0	0	0	0	0	0	0	11	6	5	0	2	0	3
150	TMUE_s0009003900	U1 small nuclear ribonucleoprotein C	84	6	4	3	5	5	3	3	0	0	0	0	0	0	0	0	0	0
151	TMUE_s0022005500	eukaryotic translation elongation factor 1 delta	21	0	0	0	0	0	0	0	0	0	4	4	5	4	5	4	4	0
152	TMUE_s0078002100	conserved hypothetical protein	41	3	0	4	3	4	2	0	0	0	0	6	6	5	0	0	0	0
153	TMUE_s0121003300	beta hexosaminidase subunit beta	48	6	3	4	3	0	2	8	0	0	0	0	0	0	0	0	0	0
154	TMUE_s0057001700	WAP-domain containing protein	26	0	0	0	0	0	0	0	0	0	0	0	5	8	0	5	2	6
155	TMUE_s0051007100	propionyl coenzyme A carboxylase beta chain	60	0	0	2	5	11	5	3	0	0	0	0	0	0	0	0	0	0
156	TMUE_s0103004600	conserved hypothetical protein	20	5	4	2	3	4	0	2	0	0	0	0	0	0	0	2	2	3
157	TMUE_s0293000700	hypothetical protein	23	0	0	0	0	0	0	0	0	2	3	5	4	5	0	5	3	3

158	TMUE_s0007005500	peroxiredoxin 2	121	3	4	4	3	4	4	6	0	0	0	0	0	0	0	0	0	0
159	TMUE_s0006004800	nucleoside diphosphate kinase	19	0	0	0	0	0	0	0	0	2	7	5	4	2	0	0	0	0
160	TMUE_s0037006300	protein unc	808	0	3	4	0	0	0	0	0	0	0	0	0	0	0	0	0	0
161	TMUE_s0064002600	hypothetical protein	18	0	0	0	0	0	0	0	0	0	0	2	0	2	2	5	2	2
162	TMUE_s0037006000	thioredoxin	21	0	0	0	0	4	2	0	0	0	0	0	2	2	4	5	3	4
163	TMUE_s0004017800	porin	41	0	0	0	0	0	0	0	0	0	2	5	4	3	4	3	2	2
164	TMUE_s0147001700	hypothetical protein	15	8	5	6	3	4	2	0	0	0	0	0	0	0	0	0	0	0
165	TMUE_s0148001700	Bravo FIGEY and I-set and fn3 and Ig 2-domain conta	141	2	7	5	3	3	4	6	0	0	0	0	0	0	0	0	0	0
166	TMUE_s0024001300	TSP 1 and Reeler and Spond N and Peptidase M23 a	124	8	6	0	3	3	3	2	0	0	0	0	0	0	0	0	0	0
167	TMUE_s0003017400	Rab GDP dissociation inhibitor alpha	48	5	6	5	3	4	0	2	0	0	0	0	0	0	0	0	0	0
168	TMUE_s0004006600	OV 16 antigen	23	0	0	0	0	0	0	0	0	0	0	0	0	0	2	8	7	7
169	TMUE_s0019001800	conserved hypothetical protein	113	0	0	0	0	0	0	0	2	0	0	0	0	2	0	0	0	2
170	TMUE_s0074001100	hypothetical protein	53	0	0	0	0	0	0	0	0	0	4	6	3	5	0	2	2	0
171	TMUE_s0022009800	hypothetical protein	141	0	0	0	0	0	0	0	2	0	0	0	0	0	0	4	2	2
172	TMUE_s0004003800	hypothetical protein	29	0	0	0	0	0	0	0	0	0	0	0	2	5	6	7	5	2
173	TMUE_s0042005600	Motile Sperm-domain containing protein	24	0	0	0	0	0	0	0	7	8	5	5	5	4	0	0	0	0
174	TMUE_s0066000700	iron dependent peroxidase	37	0	0	0	0	0	0	0	4	5	6	5	4	0	0	0	0	0
175	TMUE_s0111002000	fatty acid binding protein	28	0	0	0	0	0	0	0	0	0	0	0	0	0	4	7	7	6
176	TMUE_s0002011300	hypothetical protein	29	0	2	3	0	0	0	0	0	0	0	0	0	0	3	4	4	6
177	TMUE_s0115000200	ATP dependent RNA helicase abstrakt	250	0	0	0	0	0	0	0	0	0	3	0	0	2	2	0	0	0
178	TMUE_s0104003200	NUC194 and PI3 PI4 kinase and FATC-domain conta	442	0	0	0	0	0	0	0	3	0	0	3	3	0	0	0	0	0
179	TMUE_s0242001400	histone H2B, gonadal	24	4	4	5	5	4	0	0	0	0	0	0	0	0	0	0	0	0
180	TMUE_s0004005300	Trypsin-domain containing protein	64	0	0	0	0	0	0	0	10	9	3	5	2	5	0	0	0	0
181	TMUE_s0115002500	midasin	563	0	0	2	0	3	0	0	0	0	0	3	0	0	0	0	0	0
182	TMUE_s0054003300	Spectrin alpha chain	315	0	0	0	0	0	0	0	0	4	2	0	0	2	3	0	0	0
183	TMUE_s0042008400	Peptidase M2-domain containing protein	40	6	4	5	3	4	2	2	0	0	0	0	0	0	0	0	0	0
184	TMUE_s0005007100	protein mig c; protein mig b; protein mig a	234	0	0	0	0	0	0	0	0	0	0	0	0	6	2	7	5	10
185	TMUE_s0028009500	CBM 14-domain containing protein	195	6	4	5	4	2	0	4	0	0	0	0	0	0	0	0	0	0
186	TMUE_s0012010600	EGF-domain containing protein	78	0	0	0	0	0	0	0	8	8	3	5	0	2	0	0	0	0
187	TMUE_s0085004400	H3 histone, family 3A	21	4	4	0	7	9	3	0	0	0	0	0	0	0	0	0	0	0
188	TMUE_s0328000400	hypothetical protein	19	4	4	0	0	3	0	0	0	0	0	0	0	4	0	2	0	0
189	TMUE_s0016007600	hypothetical protein	23	2	0	0	0	0	0	0	0	0	3	8	5	4	0	4	0	0
190	TMUE_s0106000600	Moesin:ezrin:radixin 1	69	8	5	7	5	4	0	0	0	0	0	0	0	0	0	0	0	0
191	TMUE_s0038007500	DDE Tnp IS1595 and Pfam-B 9093-domain containin	25	7	3	5	0	3	0	4	0	0	0	0	0	0	0	0	0	0
192	TMUE_s0208001500	CAP-domain containing protein	30	10	0	0	0	0	0	5	0	0	0	0	0	0	0	0	0	0
193	TMUE_s0015011200	protein unc g; protein unc f; protein unc d; protein unc	761	7	5	0	0	0	0	0	0	0	0	0	0	0	0	0	0	0
194	TMUE_s0053003800	Trypsin-domain containing protein	33	0	0	0	0	0	0	0	3	2	6	3	7	3	0	2	0	0
195	TMUE_s0025005200	Trypsin-domain containing protein	84	0	0	0	0	0	0	0	9	3	0	0	5	5	0	4	0	0
196	TMUE_s0043002900	hypothetical protein	9	0	0	2	0	3	0	0	0	0	0	0	0	0	0	0	4	2
197	TMUE_s0045002900	serine protease inhibitor Kazal type 4	8	0	0	0	0	0	0	0	0	0	2	4	2	3	0	3	7	4
198	TMUE_s0009012600	SapB 2 and SapB 1 and Pfam-B 4130-domain contair	58	2	4	5	3	6	2	5	0	0	0	0	0	0	0	0	0	0
199	TMUE_s0243000200	WAP-domain containing protein	29	0	0	0	0	0	0	0	0	0	0	0	0	2	5	8	5	4
200	TMUE_s0167001400	porin	25	0	0	0	0	0	0	0	3	3	5	2	2	0	0	2	2	0
201	TMUE_s0103000900	glutathione S transferase	23	0	0	0	0	0	0	0	0	0	0	0	9	5	2	2	2	2
202	TMUE_s0117003000	eukaryotic translation elongation factor 1A	49	4	4	4	5	3	3	3	0	0	0	0	0	0	0	0	0	0
203	TMUE_s0014006600	Motile Sperm-domain containing protein	15	6	5	2	2	4	0	5	0	0	0	0	0	0	0	0	0	0
204	TMUE_s0060004900	DUF1759 and rve and DUF1758 and RVT 1 and Pept	192	0	5	5	0	0	0	0	0	0	0	0	0	0	0	0	0	0
205	TMUE_s0137000900	conserved hypothetical protein	39	3	2	2	2	0	0	0	0	0	0	2	3	3	0	2	0	0
206	TMUE_s0167000500	Whey acidic protein	16	0	0	0	0	0	0	0	0	0	13	13	18	8	5	5	0	0
207	TMUE_s0010002900	calreticulin	76	0	2	0	0	0	0	0	7	8	0	0	0	0	0	0	0	0
208	TMUE_s0012011300	neurogenic locus notch protein 2	103	0	0	0	0	0	0	0	0	2	2	4	2	2	0	0	0	0
209	TMUE_s0071007100	cathepsin F	55	0	0	0	0	0	0	0	0	3	5	5	0	3	0	0	0	0
210	TMUE_s0050007000	dynein heavy chain	563	0	0	0	0	0	0	0	0	0	0	0	0	0	4	0	0	3
211	TMUE_s0087001400	nucleobindin 1	67	0	0	0	0	0	0	0	0	0	2	2	2	3	0	3	5	4
212	TMUE_s0141002100	Actin depolymerizing factor 1, a:b	19	0	0	0	0	0	0	0	0	0	0	0	2	5	2	7	0	0
213	TMUE_s0095004900	Pkinase and TRAPP-domain containing protein	205	0	0	0	0	0	0	0	0	0	0	0	3	0	0	5	0	0
214	TMUE_s0020003500	conserved hypothetical protein	123	0	0	0	0	2	0	0	3	2	0	2	0	0	0	0	0	0
215	TMUE_s0227000900	disorganized muscle protein 1	31	0	0	0	0	0	0	0	4	3	5	2	3	4	0	2	0	0
216	TMUE_s0005016100	peptidyl prolyl cis trans isomerase FKBP4	52	0	0	0	0	0	0	0	0	3	4	4	6	3	0	0	0	0
217	TMUE_s0197001000	multiple epidermal growth factor domains	113	0	0	2	0	4	8	6	0	3	0	0	0	0	0	0	0	0
218	TMUE_s0059002100	Spectrin beta chain	266	0	0	0	0	0	0	0	0	0	0	0	4	0	0	2	0	0
219	TMUE_s0003011400	Cadherin and EGF CA and Laminin G 2-domain conta	499	0	0	0	0	0	0	0	0	0	0	0	0	0	3	2	0	0
220	TMUE_s0223000400	phosphatidylethanolamine binding protein	17	0	0	0	0	0	0	0	0	0	0	0	0	0	3	8	4	3
221	TMUE_s0044005700	Pfam-B 4743 and Pfam-B 7628-domain containing pr	386	0	0	0	0	0	0	0	0	0	0	2	2	3	3	0	0	2
222	TMUE_s0051004900	sulfhydryl oxidase 1	70	0	0	0	0	0	0	0	0	0	0	6	5	0	0	2	0	0
223	TMUE_s0016007700	hypothetical protein	11	0	0	0	0	0	0	0	0	0	0	0	0	2	3	7	6	4
224	TMUE_s0210000100	Vacuolar protein sorting associated protein 13A	369	0	0	0	0	0	0	0	0	0	0	0	0	0	2	0	0	2
225	TMUE_s0069001000	Trypsin-domain containing protein	62	0	0	0	0	0	0	0	8	12	6	0	0	0	0	0	0	0
226	TMUE_s0041004600	26S proteasome non ATPase regulatory subunit 10	27	0	0	0	0	0	0	0	0	4	2	0	3	0	2	4	2	2
227	TMUE_s0009009900	protein asteroid	28	2	0	0	0	0	0	2	0	0	0	0	0	3	2	2	3	3
228	TMUE_s0003007500	coagulation factor IX	15	0	0	0	0	0	0	0	2	4	7	4	3	0	0	0	3	0
229	TMUE_s0013012700	Motile Sperm-domain containing protein	14	0	0	0	0	0	0	0	0	0	0	2	4	7	5	4	2	0
230	TMUE_s0004011200	T complex protein 1 subunit zeta	358	0	0	0	0	0	0	0	0	0	0	3	0	0	0	2	0	0
231	TMUE_s0115002700	Prefoldin and WAP-domain containing protein	60	6	5	4	4	0	2	0	0	0	0	0	0	0	0	0	0	0
232	TMUE_s0059005200	reverse transcriptase	31	0	0	0	0	0	0	0	0	0	0	0	0	2	0	2	3	2
233	TMUE_s0001018700	hydroxyacylglutathione hydrolase	30	0	0	0	0	0	0	0	0	0	0	0	0	4	5	7	0	0
234	TMUE_s0076002300	Proteasome subunit beta type 5	31	0	3	5	5	2	2	0	0	0	0	0	0	0	0	0	0	0
235	TMUE_s0069001100	Pfam-B 3281 and Trypsin-domain containing protein	71	0	0	0	0	0	0	0	12	4	2	0	0	0	0	0	0	0
236	TMUE_s0213000400	serine protease	50	0	0	0	0	0	0	0	0	0	0	0	0	0	0	6	3	5
237	TMUE_s0105000400	myoglobin	24	0	0	0	0	0	0	0	0	0	0	0	0	2	2	5	2	2
238	TMUE_s0020006600	tumor necrosis factor alpha induced protein	49	0	0	0	0	0	0	0	0	0	2	0	3	0	2	2	2	3
239	TMUE_s0071001300	stress induced phosphoprotein 1	59	0	0	0	0	0	0	0	0	2	0	3	4	3	0	3	0	0
240	TMUE_s0117000900	transmembrane serine protease 8	36	5	5	3	2	3	0	0	0	0	0	0	0	0	0	0	0	0
241	TMUE_s0021000500	neurofibromin	321	0	3	0	0	0	0	0	0	3	2	0	0	0	0	0	0	0
242	TMUE_s0118001200	Glyco hydro 18 and CBM 14-domain containing prote	81	4	4	5	4	4	4	0	0	0	0	0	0	0	0	0	0	0
243	TMUE_s0029002700	L lactate dehydrogenase	90	0	0	0	0	0	0	0	3	3	3	0	0	0	2	0	0	0
244	TMUE_s0203000700	Pfam-B 2117-domain containing protein	223	0	0	0	2	7	0	0	0	0	0	0	0	0	0	0	0	0
245	TMUE_s0016003600	methionine tRNA ligase, cytoplasmic	125	0	0	0	0	0	0	0	4	2	0	0	0	0	0	0	0	0
246	TMUE_s0046002300	hypothetical protein	107	0	0	0	0	0	0	0	2	2	0	0	0	0	0	2	0	0
247	TMUE_s0008014300	hypothetical protein	20	0	0	0	0	0	0	0	0	2	0	0	0	2	0	0	0	0
248	TMUE_s0010001600	inositol 1,4,5 trisphosphate receptor type 1	296	0	0	5	0	0	0	3	0	0	0	0	0	0	0	0	0	0
249	TMUE_s0048003700	E3 ubiquitin protein ligase HUWE1	459	0	0	0	0	0	0	0	0	0	2	0	2	0	0	0	0	0
250	TMUE_s0239000100	pyruvate kinase	67	3	6	4	0	3	0	0	0	0	0	0	0	0	0	0	0	0
251	TMUE_s0122001300	Cystatin-domain containing protein	13	0	0	0	0	0	0	0	0	0	0	0	0	0	0	7	5	4
252	TMUE_s0115001600	WAP-domain containing protein	24	0	0	0	0	0	0	0	0	0	0	0	4	4	2	4	2	2
253	TMUE_s0172001800	CH-domain containing protein	41	5	0	2	3	3	3	0	0	0	0	0	0	0	0	0	0	0
254	TMUE_s0003001900	serine proteinase inhibitor	47	3	4	3	0	4	0	2	0	0	0	0	0	0	0	0	0	0
255	TMUE_s0037007000	Pfam-B 927 and Beach and WD40-domain containing	369	0	0	0	0	0	0	0	0	0	0	3	0	3	0	0	0	0
256	TMUE_s0083000200	inorganic pyrophosphatase	63	0	0	0	0	0	0	0	0	2	5	6	3	0	0	0	0	0
257	TMUE_s0011009300	zf-B box and Pfam-B 17973 and AIP3-domain contain	104	0	0	0	3	0	3	0	0	0	0	0	0	0	0	0	0	0
258	TMUE_s0040003900	muscle cell intermediate filament protein OV71	68	0	0	0	0	0	0	0	0	0	0	0	0	0	2	5	2	5
259	TMUE_s0026007800	chaperonin protein heat shock protein60	52	0	0	0	0	0	0	0	0	0	0	0	0	0	0	6	0	3
260	TMUE_s0075004300	RETRotransposon family member	188	4	8	3	4	6	0	0	0	0	0	0	0	0	0	0	0	0
261	TMUE_s0014014200	proteasome subunit alpha type 1	29	0	3	4	6	4	0	0	0	0	0	0	0	0	0	0	0	0
262	TMUE_s0018001600	sodium:potassium transporting ATPase subunit	71	2	4	0	2	0	0	0	0	0	0	0	0	0	0	0	0	0
263	TMUE_s0203000200	transmembrane protein mitochondrial	34	0	0	0	2	2	2	0	0	0	0	0	0	0	0	2	0	0
264	TMUE_s0085005000	venom allergen 5	39	0	0	0	0	0	0	0	0	0	3	5	0	2	0	3	0	0
265	TMUE_s0104001300	hypothetical protein	10	2	0	0	0	0	2	3	0	0	0	0	0	0	0	0	0	0
266	TMUE_s0078002700	His Phos 2-domain containing protein	52	0	5	3	4	2	2	2	0	0	0	0	0	0	0	0	0	0
267	TMUE_s0064001200	Pfam-B 9093-domain containing protein	38	0	0	0	0	0	0	0	0	0	0	6	6	5	0	2	0	3
268	TMUE_s0012007900	Troponin and Pfam-B 969-domain containing protein	83	0	0	0	0	0	0	0	4	4	2	2	2	0	0	0	0	0
269	TMUE_s0156000300	Exo endo phos-domain containing protein	50	0	0	0	0	0	0	0	5	6	0	3	3	2	0	0	0	0
270	TMUE_s0023004500	Pfam-B 9093-domain containing protein	39	4	2	3	4	2	0	0	0	0	0	0	0	0	0	0	0	0
271	TMUE_s0089003300	DB module family protein	24	0	0	0	0	0	0	0	0	0	0	0	0	0	0	7	4	6
272	TMUE_s0116002200	ubiquitin associated and SH3	41	6	4	2	3	0	0	0	0	0	0	0	0	0	0	0	0	0
273	TMUE_s0018008000	CBM 14-domain containing protein	78	0	0	0	4	10	7	2	0	0	0	0	0	0	0	0	0	0
274	TMUE_s0018008800	collagen alpha (iv) chain	202	3	3	0	0	0	0	0	0	0	0	0	0	0	0	0	0	0
275	TMUE_s0047004200	pathogeneis protein 1B	46	4	2	2	0	4	0	0	0	0	0	0	0	0	0	0	0	2
276	TMUE_s0141002000	actin depolymerizing factor 2 c	20	0	0	0	0	0	0	0	0	0	0	0	0	2	2	6	2	0
277	TMUE_s0102004000	hypothetical protein	30	0	0	0	0	0	0	0	0	0	3	3	4	0	0	0	0	0
278	TMUE_s0006008000	AdoHcyase and AdoHcyase NAD-domain containing	49	3	2	2	2	0	2	4	0	0	0	0	0	0	0	0	0	0
279	TMUE_s0096005200	ubiquitin associated and SH3	39	5	5	3	5	2	2	0	0	0	0	0	0	0	0	0	0	0
280	TMUE_s0001010500	Pfam-B 7199-domain containing protein	101	0	0	0	0	0	0	0	0	0	2	0	0	2	0	0	0	0
281	TMUE_s0011008400	dynein heavy chain	447	3	3	0	2	0	0	0	0	0	0	0	0	0	0	0	0	0
282	TMUE_s0009001300	translationally controlled tumor protein	21	0	0	0	0	0	0	0	0	0	0	6	5	4	0	2	0	0
283	TMUE_s0049002800	Pfam-B 1741-domain containing protein	188	0	0	0	3	7	3	2	0	0	0	0	0	0	0	0	0	0
284	TMUE_s0023000500	Pfam-B 9093-domain containing protein	26	0	0	0	0	0	0	0	0	0	6	6	3	4	0	0	0	0
285	TMUE_s0106001000	Pfam-B 6613 and Laminin G 2-domain containing pro	165	0	0	3	3	2	0	2	0	0	0	0	0	0	0	0	0	0
286	TMUE_s0072003400	independent phosphoglycerate mutase	46	0	0	0	0	0	0	0	0	0	0	4	3	4	0	0	0	0
287	TMUE_s0078002500	Troponin-domain containing protein	40	0	0	0	0	0	0	0	5	5	4	0	0	0	0	0	0	0
288	TMUE_s0119002100	galectin	31	2	0	0	0	0	0	0	0	0	0	0	0	5	3	3	0	0
289	TMUE_s0044001400	conserved hypothetical protein	61	2	0	0	0	2	0	0	4	3	0	0	0	0	0	0	0	0
290	TMUE_s0061004400	conserved hypothetical protein	111	0	0	0	2	0	0	0	0	0	0	0	0	0	0	0	0	2
291	TMUE_s0130000500	cathepsin D aspartic protease	47	4	2	3	3	0	0	3	0	0	0	0	0	0	0	0	0	0
292	TMUE_s0116004800	poly(U) specific endoribonuclease	33	0	0	0	0	0	0	0	0	10	4	3	0	0	0	0	0	0
293	TMUE_s0094002300	Pfam-B 2610 and Trypsin and MMR HSR1-domain cc	143	0	0	0	0	0	0	0	0	2	3	3	0	2	0	0	0	0
294	TMUE_s0012000200	conserved hypothetical protein	148	0	0	0	2	0	0	0	0	0	0	0	0	2	0	0	2	0
295	TMUE_s0007006700	chymotrypsin inhibitor	15	0	0	0	0	0	0	0	0	0	0	0	0	0	0	0	4	6
296	TMUE_s0005001100	Kunitz protease inhibitor	25	0	0	0	0	0	0	0	0	0	0	0	0	0	2	2	4	0
297	TMUE_s0213000300	serine protease	58	4	0	4	3	3	3	0	0	0	0	0	0	0	0	0	0	0
298	TMUE_s0005013600	alpha mannosidase 2C1	117	3	2	2	4	3	0	0	0	0	0	0	0	0	0	0	0	0
299	TMUE_s0037004300	hypothetical protein	58	0	0	0	0	0	0	0	4	10	0	0	0	0	0	0	0	0
300	TMUE_s0036005400	Neutral alpha glucosidase AB	202	0	0	0	0	0	0	0	9	5	0	0	0	0	0	0	0	0
301	TMUE_s0123000300	protein ketn d; protein ketn c; protein ketn b; protein ke	1721	0	0	5	0	6	0	0	0	0	0	0	0	0	0	0	0	0
302	TMUE_s0018010700	DUF1759-domain containing protein	140	0	0	6	7	0	0	0	0	0	0	0	0	0	0	0	0	0
303	TMUE_s0106000600	Moesin:ezrin:radixin 1	69	0	0	0	0	0	0	0	0	2	0	0	0	3	0	0	0	0
304	TMUE_s0010006100	calmodulin	18	0	0	0	0	0	0	0	0	0	2	2	4	3	0	2	0	0
305	TMUE_s0098000400	protein wos2	21	0	0	0	0	0	0	0	0	0	0	4	0	2	0	3	0	0
306	TMUE_s0028000600	VWA-domain containing protein	44	0	0	0	0	0	0	0	4	4	2	2	3	0	0	0	0	0
307	TMUE_s0058002200	protein tag 76	99	0	0	0	0	0	0	0	0	2	0	2	0	0	0	0	0	0
308	TMUE_s0077000200	hypothetical protein	104	0	0	0	0	0	0	0	0	2	2	0	0	0	0	0	0	0
309	TMUE_s0014004300	protein lap4	300	0	0	0	0	0	0	0	0	0	0	2	0	3	0	0	0	0
310	TMUE_s0204000100	Motile Sperm-domain containing protein	21	2	0	0	0	2	5	7	0	0	0	0	0	0	0	0	0	0
311	TMUE_s0086002000	Methyltransf 31-domain containing protein	39	0	0	0	0	0	0	0	0	5	5	4	2	0	0	0	0	0
312	TMUE_s0018008500	polyadenylate binding protein 1	79	0	0	0	0	0	0	0	0	0	0	0	0	2	3	2	0	0
313	TMUE_s0062004700	ubiquitin-domain containing protein	95	0	0	0	0	0	0	0	0	0	0	0	0	0	0	0	7	8
314	TMUE_s0049001600	Trypsin-domain containing protein	215	0	0	0	0	0	0	0	10	8	0	0	0	0	0	0	0	0
315	TMUE_s0050003800	carboxypeptidase e	232	0	0	2	3	5	0	0	0	0	0	0	0	0	0	0	0	0
316	TMUE_s0133001900	heat shock 70 kDa protein 4	95	0	0	0	0	0	0	0	5	8	2	0	0	0	0	0	0	0
317	TMUE_s0009000200	pathogeneis protein 1B	19	0	0	0	0	0	0	0	0	0	0	2	2	2	4	2	0	0
318	TMUE_s0184000700	conserved hypothetical protein	37	0	4	2	0	0	0	0	0	0	0	0	0	0	0	0	0	0

319	TMUE_s0019001900	DUF21-domain containing protein	63	0	0	0	0	0	0	0	0	0	0	3	2	0	0	2	0	0
320	TMUE_s0049001700	hypothetical protein	23	0	0	0	0	0	0	0	0	0	2	3	3	3	0	0	0	0
321	TMUE_s0018003300	proteasome (prosome, macropain) subunit, beta	25	3	0	4	3	2	3	0	0	0	0	0	0	0	0	0	0	0
322	TMUE_s0027001900	hypothetical protein	50	0	0	0	0	0	0	0	0	0	0	0	3	4	0	0	0	0
323	TMUE_s0089001100	conserved hypothetical protein	54	0	0	0	0	0	0	0	2	7	3	3	0	0	0	0	0	0
324	TMUE_s0016007800	hypothetical protein	10	0	0	0	0	0	0	0	0	0	0	0	0	0	2	5	5	3
325	TMUE_s0012010900	Neurogenic locus notch protein	43	0	0	0	0	0	0	0	0	6	4	0	0	0	0	0	0	0
326	TMUE_s0015003400	nuclear migration protein nudC	39	0	0	0	0	0	0	0	0	0	0	4	0	0	3	0	2	0
327	TMUE_s0018004100	hypothetical protein	29	0	0	0	0	0	0	0	0	0	0	0	2	0	0	3	0	0
328	TMUE_s0018011800	WAP type 'four disulfide core'	26	0	0	0	0	0	0	0	0	0	0	0	3	3	0	4	0	0
329	TMUE_s0116003000	adenylate kinase isoenzyme 1	20	6	4	3	4	3	0	0	0	0	0	0	0	0	0	0	0	0
330	TMUE_s0014008700	Tyrosinase and HTH 9 and Pfam-B 8516-domain con	104	0	0	0	0	0	0	0	0	2	2	0	0	0	0	0	0	0
331	TMUE_s0032002000	hypothetical protein	162	0	0	0	2	0	0	0	0	0	2	0	0	0	0	0	0	0
332	TMUE_s0008004200	histone H2A	14	0	0	0	0	0	0	0	3	0	0	0	0	0	0	0	2	0
333	TMUE_s0064002700	hypothetical protein	27	2	0	0	0	2	0	0	0	0	0	0	2	2	0	2	0	0
334	TMUE_s0125002500	Cupin 8-domain containing protein	59	3	0	5	0	2	2	2	0	0	0	0	0	0	0	0	0	0
335	TMUE_s0018000600	DUF290-domain containing protein	15	0	0	0	0	0	0	0	0	0	0	0	3	9	2	0	2	0
336	TMUE_s0058001000	Trypsin-domain containing protein	47	0	0	0	0	0	0	0	0	0	2	3	3	0	0	0	0	0
337	TMUE_s0019005400	tropomyosin	34	0	0	0	4	0	0	0	6	0	0	0	0	0	0	0	0	0
338	TMUE_s0014009000	low density lipoprotein receptor protein	112	0	0	0	0	0	0	0	0	0	0	0	0	2	0	2	0	0
339	TMUE_s0275000100	neuron navigator 2	205	0	0	0	0	0	0	0	0	0	0	0	2	2	0	0	0	0
340	TMUE_s0149000500	intraflagellar transport protein 172	240	0	0	0	0	0	0	0	0	0	0	0	2	0	3	0	0	0
341	TMUE_s0008012900	Peptidase M14-domain containing protein	47	0	0	0	0	0	0	0	0	0	3	0	2	2	0	0	0	0
342	TMUE_s0146001900	Trypsin-domain containing protein	52	0	0	0	0	0	0	0	0	0	2	5	3	2	0	0	0	0
343	TMUE_s0134002300	valyl trna synthetase; valine trna ligase	108	2	2	2	0	0	0	0	0	0	0	0	0	0	0	0	0	0
344	TMUE_s0033004500	RUN and PDZ and Pfam-B 1902 and zf-CCCH and Pf	202	0	0	0	0	0	0	0	0	0	0	0	0	0	2	3	0	0
345	TMUE_s0162002000	Pfam-B 11267 and gag pre-integrs and rve and DUF4	288	0	0	0	0	0	0	0	0	0	0	0	0	3	2	0	0	0
346	TMUE_s0003012200	glutamine oxaloacetic transaminase	49	0	0	0	0	0	0	0	3	6	6	0	0	0	0	0	0	0
347	TMUE_s0134000700	plastin 3	76	0	0	0	0	0	0	0	0	0	0	2	3	0	0	0	0	0
348	TMUE_s0207000500	laminin subunit beta 1	218	4	3	2	2	0	0	0	0	0	0	0	0	0	0	0	0	0
349	TMUE_s0028006300	sb:cb283 protein	57	0	0	0	0	4	6	0	0	0	0	0	0	0	0	0	0	0
350	TMUE_s0185001200	hypothetical protein	22	2	0	0	0	0	0	0	0	0	0	3	3	0	0	0	0	0
351	TMUE_s0155001100	conserved hypothetical protein	40	0	0	0	0	2	3	0	2	0	0	0	0	0	0	0	0	0
352	TMUE_s0004012300	LIM-domain containing protein	24	0	0	0	2	0	0	0	0	0	0	0	0	0	0	2	0	2
353	TMUE_s0078001400	DUF290-domain containing protein	11	0	0	0	0	0	0	0	0	0	3	4	6	0	0	0	0	0
354	TMUE_s0004019200	Mononeg RNA pol-domain containing protein	19	0	0	2	0	4	0	0	0	0	0	0	0	0	0	0	0	0
355	TMUE_s0051006000	regulating synaptic membrane exocytosis 2	174	0	0	3	2	5	0	0	0	0	0	0	0	0	0	0	0	0
356	TMUE_s0002020500	copia protein	142	0	0	0	0	0	0	0	0	0	0	0	3	0	0	2	0	0
357	TMUE_s0004009900	Heterogeneous nuclear ribonucleoprotein A1	38	0	0	0	0	0	0	0	0	0	0	0	3	2	0	0	2	2
358	TMUE_s0052004200	plasma alpha l fucosidase	52	3	5	0	0	2	2	0	0	0	0	0	0	0	0	0	0	0
359	TMUE_s0040005200	troponin C	19	0	0	0	0	0	0	0	6	4	3	3	2	0	0	0	0	0
360	TMUE_s0005014100	hunchback transcription factor	304	0	0	0	4	0	2	0	0	0	0	0	0	0	0	0	0	0
361	TMUE_s0060007700	proteasome subunit beta type 1	27	0	0	3	3	3	0	2	0	0	0	0	0	0	0	0	0	0
362	TMUE_s0042006800	retrovirus Pol polyprotein	151	0	0	0	0	0	0	0	0	0	2	0	0	2	0	0	0	0
363	TMUE_s0036003100	nucleosome assembly protein 1 1	38	0	0	2	0	4	5	4	0	0	0	0	0	0	0	0	0	0
364	TMUE_s0044004800	DNase II-domain containing protein	42	0	0	0	0	0	0	0	0	0	2	2	4	2	0	0	0	0
365	TMUE_s0012008900	hypothetical protein	13	0	0	0	0	0	0	0	0	0	0	0	0	0	0	2	0	2
366	TMUE_s0023004600	Pfam-B 9093-domain containing protein	27	0	3	2	2	3	0	0	0	0	0	0	0	0	0	0	0	0
367	TMUE_s0155002600	Astacin and MH2-domain containing protein	101	2	2	0	3	0	0	0	0	0	0	0	0	0	0	0	0	0
368	TMUE_s0043001100	charged multivesicular body protein 1b	22	0	3	4	2	6	0	0	0	0	0	0	0	0	0	0	0	0
369	TMUE_s0086001800	transient receptor potential cation channel	169	0	3	0	0	0	2	3	0	0	0	0	0	0	0	0	0	0
370	TMUE_s0004007100	Trypsin-domain containing protein	107	0	0	0	0	0	0	0	0	0	0	0	0	0	0	6	0	6
371	TMUE_s0175001100	E3 SUMO protein ligase RanBP2	235	0	0	0	0	0	0	0	0	0	0	2	0	2	0	0	0	0
372	TMUE_s0007004700	conserved hypothetical protein	17	0	0	2	0	0	0	0	0	0	0	0	2	2	2	0	0	0
373	TMUE_s0006006600	mediator of RNA polymerase II transcription	74	0	0	0	0	0	0	0	0	0	0	0	0	3	3	0	0	2
374	TMUE_s0041006400	hypothetical protein	33	0	2	0	0	0	0	0	0	0	0	0	0	0	0	4	0	0
375	TMUE_s0007008400	Gamma interferon inducible lysosomal thiol	30	2	4	2	2	4	0	0	0	0	0	0	0	0	0	0	0	0
376	TMUE_s0038000700	cullin 1	94	0	0	0	0	0	0	0	0	0	0	0	2	0	0	2	0	0
377	TMUE_s0122001100	pol polyprotein	43	3	2	0	4	0	0	3	0	0	0	0	0	0	0	0	0	0
378	TMUE_s0023003100	hypothetical protein	83	0	4	0	0	0	0	0	0	0	0	3	0	0	0	0	0	0
379	TMUE_s0003008700	trans 2,3 enoyl coenzyme A reductase	64	0	0	0	0	0	0	0	0	0	0	0	0	0	0	3	2	2
380	TMUE_s0061001100	DNA polymerase epsilon catalytic subunit A	245	0	5	3	0	0	2	0	0	0	0	0	0	0	0	0	0	0
381	TMUE_s0269000500	phospholipase B 2	70	0	0	0	0	4	4	4	0	0	0	0	0	0	0	0	0	0
382	TMUE_s0064002100	hypothetical protein	14	0	0	0	0	0	0	0	0	0	0	0	0	4	0	5	0	0
383	TMUE_s0177000300	carbonic anhydrase 2	36	0	0	0	0	0	0	0	4	2	2	0	0	0	0	0	0	0
384	TMUE_s0355000600	Unknown (protein for IMAGE:7068038)	28	0	0	0	0	0	0	0	0	0	0	0	7	2	0	0	0	0
385	TMUE_s0028004300	hypothetical protein	81	0	0	0	0	0	0	0	0	0	0	2	2	0	0	0	0	0
386	TMUE_s0137001700	macrophage migration inhibitory factor	24	0	0	0	0	0	0	0	0	0	0	0	0	0	3	3	2	3
387	TMUE_s0031003000	calsequestrin family protein	47	2	2	3	0	0	2	0	0	0	0	0	0	0	0	0	0	0
388	TMUE_s0039005000	leucine rich repeat serine:threonine protein	278	0	0	0	0	0	0	0	0	3	2	0	0	0	0	0	0	0
389	TMUE_s0053000300	ryanodine receptor	574	0	0	3	0	0	2	2	0	0	0	0	0	0	0	0	0	0
390	TMUE_s0043002300	Trypsin-domain containing protein	33	0	0	0	0	0	0	0	0	0	0	0	0	2	0	2	2	0
391	TMUE_s0013011500	Proteasome subunit alpha type 2	25	0	3	5	3	4	0	0	0	0	0	0	0	0	0	0	0	0
392	TMUE_s0003012900	BTB-domain containing protein	134	0	0	0	0	0	0	0	0	2	0	0	0	0	0	0	2	0
393	TMUE_s0052005500	paramyosin	103	0	0	2	0	3	0	0	0	0	0	0	0	0	0	0	0	0
394	TMUE_s0103004000	glutaminyl tRNA synthetase	205	0	0	0	0	0	0	0	0	0	0	2	0	0	3	0	0	0
395	TMUE_s0039004800	Proteasome subunit alpha type 4	48	0	2	2	2	0	0	0	0	0	0	0	0	0	0	0	0	0
396	TMUE_s0069005100	26S proteasome non ATPase regulatory subunit 10	152	3	2	0	0	0	0	0	0	0	0	0	0	0	0	0	0	0
397	TMUE_s0079003500	Intermediate filament protein ifa 1	71	0	0	0	0	4	4	0	0	0	0	0	0	0	0	0	0	0
398	TMUE_s0014005900	procollagen lysine,2 oxoglutarate 5 dioxygenase	116	3	0	3	0	0	0	0	0	0	0	0	0	0	0	0	0	0
399	TMUE_s0145002500	JNK interacting protein	170	0	0	0	0	0	0	0	2	0	0	0	0	0	0	0	0	3
400	TMUE_s0029000200	xaa Pro aminopeptidase 1	69	0	0	0	0	0	0	0	9	5	0	0	0	0	0	0	0	0
401	TMUE_s0008017400	hypothetical protein	13	0	2	2	0	0	0	0	0	0	0	0	0	0	0	4	0	0
402	TMUE_s0016009700	WAP-domain containing protein	34	0	0	0	0	0	0	0	0	0	0	2	3	0	0	2	0	2
403	TMUE_s0319000400	hypothetical protein	32	0	0	0	0	0	0	0	0	0	0	0	0	0	2	2	4	3
404	TMUE_s0104004500	Trypsin-domain containing protein	39	0	0	0	0	0	0	0	0	0	4	3	0	0	0	0	0	0
405	TMUE_s0119002700	hypothetical protein	11	0	0	0	0	0	0	0	0	0	0	0	0	2	0	2	2	0
406	TMUE_s0011007500	nuclease sensitive element binding protein 1	39	0	0	0	0	0	0	0	0	0	0	0	0	2	0	3	2	4
407	TMUE_s0030006100	UV excision repair protein RAD23 B	78	0	0	0	0	0	0	0	2	0	2	0	3	0	2	0	0	0
408	TMUE_s0004015200	intraflagellar transport protein 57	46	0	0	0	0	0	0	0	0	0	0	0	0	0	0	4	2	0
409	TMUE_s0012012300	conserved hypothetical protein	121	0	0	0	3	0	0	0	0	0	0	2	0	0	0	0	0	0
410	TMUE_s0078001100	14 kDa phosphohistidine phosphatase	13	0	0	0	0	0	0	0	0	0	0	0	0	0	0	3	3	0
411	TMUE_s0428000200	lactoylglutathione lyase	20	0	0	0	0	0	0	0	0	0	0	3	0	2	0	0	0	0
412	TMUE_s0012009200	hypothetical protein	17	0	0	0	0	0	0	0	0	0	0	0	0	4	0	3	0	0
413	TMUE_s0162000100	receptor expression enhancing protein	21	3	0	4	2	0	0	0	0	0	0	0	0	0	0	0	0	0
414	TMUE_s0136002900	mitochondrial manganese superoxide dismutase	25	0	0	0	0	0	0	0	0	0	0	0	0	0	0	4	2	3
415	TMUE_s0035000600	plexin A4	309	0	2	3	0	0	0	0	0	0	0	0	0	0	0	0	0	0
416	TMUE_s0034005800	Motile Sperm and MULE and Pfam-B 516-domain cor	41	0	0	0	0	0	0	0	0	0	0	0	0	3	3	0	0	0
417	TMUE_s0040000600	5' nucleotidase	66	0	0	0	0	0	0	0	0	13	0	6	0	0	0	0	0	0
418	TMUE_s0217000500	receptor type tyrosine protein phosphatase	275	0	0	0	0	0	0	0	4	2	0	0	2	0	0	0	0	0
419	TMUE_s0005013800	puromycin sensitive aminopeptidase	97	0	0	0	0	0	0	0	11	7	6	0	0	0	0	0	0	0
420	TMUE_s0127000900	myosin heavy chain, non muscle	233	0	0	0	0	0	0	0	0	0	0	0	2	2	3	0	0	0
421	TMUE_s0261000500	hypothetical protein	28	0	0	0	0	0	0	0	0	0	0	0	0	4	0	2	0	0
422	TMUE_s0054005700	glycogen phosphorylase	98	0	0	0	0	0	0	0	2	4	0	0	0	0	0	0	0	0
423	TMUE_s0132001200	Gut specific cysteine proteinase	39	0	0	0	0	0	0	0	0	0	0	3	2	0	0	0	0	0
424	TMUE_s0072000900	ADP ribose pyrophosphatase, mitochondrial	29	0	0	0	0	0	0	0	0	0	0	5	2	3	0	0	0	0
425	TMUE_s0102000400	carboxypeptidase O	45	2	0	2	3	0	2	0	0	0	0	0	0	0	0	0	0	0
426	TMUE_s0189000300	eukaryotic initiation factor 4A	50	0	0	0	0	0	0	0	0	0	0	2	0	0	0	2	2	0
427	TMUE_s0027006600	Asp-domain containing protein	44	3	0	5	2	0	0	0	0	0	0	0	0	0	0	0	0	0
428	TMUE_s0002013200	Phorbol ester:diacylglycerol binding protein	192	0	2	2	3	0	0	0	0	0	0	0	0	0	0	0	0	0
429	TMUE_s0092002600	wap type 'four disulfide core	26	0	0	0	0	0	0	0	0	0	0	0	0	0	0	3	2	3
430	TMUE_s0154000100	tubulointerstitial nephritis antigen	58	0	0	2	3	3	0	0	0	0	0	0	0	0	0	0	0	0
431	TMUE_s0007000900	dihydropteridine reductase	61	0	0	0	0	0	0	0	0	0	0	2	5	3	0	0	0	0
432	TMUE_s0022011000	hypothetical protein	21	3	2	0	0	0	0	0	0	0	0	0	0	0	0	0	0	0
433	TMUE_s0120003200	rve and Pfam-B 10563 and zf-CCHC and Pfam-B 254	171	0	0	0	0	0	0	0	0	3	0	0	0	0	2	0	0	0
434	TMUE_s0078001800	DUF290-domain containing protein	15	0	0	0	0	0	0	0	0	0	0	0	0	0	2	2	0	0
435	TMUE_s1008000100	hypothetical protein	26	0	0	0	2	0	2	0	0	0	0	0	0	0	0	0	0	0
436	TMUE_s0173000900	delta protein 4	54	2	0	0	0	0	0	0	2	2	0	0	0	0	0	0	0	0
437	TMUE_s0057001800	hypothetical protein	15	0	0	0	0	0	0	0	0	0	0	0	0	0	0	2	0	2
438	TMUE_s0210000600	adenylosuccinate synthetase	51	0	0	0	0	0	0	0	0	5	3	2	0	0	0	0	0	0
439	TMUE_s0036005400	Neutral alpha glucosidase AB	202	3	0	3	0	0	0	0	0	0	0	0	0	0	0	0	0	0
440	TMUE_s0006011200	transmembrane serine protease 8	38	3	0	2	3	0	0	0	0	0	0	0	0	0	0	0	0	0
441	TMUE_s0291000200	tankyrase 1	175	0	0	0	0	0	0	0	3	0	2	0	0	0	0	0	0	0
442	TMUE_s0057006300	hypoxia up regulated protein 1	103	0	0	0	0	0	0	0	2	2	0	0	0	0	0	0	0	0
443	TMUE_s0008001200	Ubiquitin carboxyl terminal hydrolase 7	160	0	2	2	0	0	0	0	0	0	0	0	0	0	0	0	0	0
444	TMUE_s0074002400	Alcohol dehydrogenase NADP+ A	37	0	0	0	0	0	0	0	0	0	0	0	6	3	2	0	0	0
445	TMUE_s0001023000	conserved hypothetical protein	72	0	0	0	2	0	0	0	0	3	0	2	0	0	0	0	0	0
446	TMUE_s0004005500	Trypsin-domain containing protein	33	0	0	0	0	0	0	0	2	0	0	2	0	0	0	0	0	0
447	TMUE_s0025002800	Gut specific cysteine proteinase	48	0	0	2	3	4	0	0	0	0	0	0	0	0	0	0	0	0
448	TMUE_s0224000200	Pfam-B 16759 and DUF3518 and Pfam-B 38 and Pfa	170	0	0	0	0	0	0	0	0	0	2	0	2	0	0	0	0	0
449	TMUE_s0156000500	WD40 and Aha1 N and AHSA1-domain containing pr	218	0	0	0	0	0	0	0	0	3	3	0	0	0	0	0	0	0
450	TMUE_s0291000100	Prominin-domain containing protein	36	4	2	0	2	0	0	0	0	0	0	0	0	0	0	0	0	0
451	TMUE_s0007010500	Peptidase A17 and DUF1759-domain containing prot	164	2	0	3	0	0	0	0	0	0	0	0	0	0	0	0	0	0
452	TMUE_s0012004300	protein unc 80	287	0	0	0	0	0	0	0	0	0	0	0	4	3	0	0	0	0
453	TMUE_s0020004300	proteasome subunit alpha type 6	29	0	2	3	2	0	2	0	0	0	0	0	0	0	0	0	0	0
454	TMUE_s0051002900	Tau tubulin kinase 1	37	0	0	0	0	0	0	0	0	0	0	0	2	4	0	0	0	0
455	TMUE_s0175000200	Pfam-B 9093-domain containing protein	37	0	11	15	0	0	0	0	0	0	0	0	0	0	0	0	0	0
456	TMUE_s0033006400	CAP-domain containing protein	35	0	0	0	0	0	0	0	22	16	0	0	0	0	0	0	0	0
457	TMUE_s0086002100	Pfam-B 11591 and Methyltransf 31-domain containing	36	3	5	3	0	0	0	0	0	0	0	0	0	0	0	0	0	0
458	TMUE_s0072004400	hsc70 interacting protein	27	0	0	0	0	0	0	0	4	2	2	2	0	0	0	0	0	0
459	TMUE_s0054003300	Spectrin alpha chain	315	0	0	0	0	2	2	0	0	0	0	0	0	0	0	0	0	0
460	TMUE_s0023002200	phosphomannomutase 2	31	0	0	0	0	0	0	0	0	2	4	4	0	0	0	0	0	0
461	TMUE_s0119001800	neprilysin 2	87	0	0	0	0	0	0	0	3	2	0	0	0	0	0	0	0	0
462	TMUE_s0006010300	Glyco hydro 38C-domain containing protein	64	0	0	0	0	0	0	0	2	2	0	0	2	0	0	0	0	0
463	TMUE_s0001023200	proteasome beta 3 subunit	23	0	0	4	0	3	0	0	0	0	0	0	0	0	0	0	0	0
464	TMUE_s0444000100	RVT 1 and Peptidase A17 and rve-domain containing	144	0	3	2	0	2	0	0	0	0	0	0	0	0	0	0	0	0
465	TMUE_s0134003400	protein sma d; protein sma c; protein sma b	469	2	5	0	0	0	0	0	0	0	0	0	0	0	0	0	0	0
466	TMUE_s0155001200	SYS1 Golgi localized integral membrane protein	55	0	0	0	0	0	0	0	7	3	0	0	0	0	0	0	0	0
467	TMUE_s0278000600	Cadherin hmr 1	132	0	0	0	0	0	0	0	4	3	0	0	0	0	0	0	0	0
468	TMUE_s0024001300	TSP 1 and Reeler and Spond N and Peptidase M23 a	124	0	0	0	0	0	0	0	0	11	7	0	0	0	0	0	0	0

Table S1. List of proteins identified within T. muris ES$^{-polycys}$. List was compiled by collating the mass spectrometry data for all the protein-containing Superose 12 fractions (fractions 16 to 32). The total spectral count is displayed for each protein in each fraction (criteria set to 95% protein threshold, 50% peptide threshold, minimum 2 peptides identified). Mw= molecular weight in kDa. Frac = fraction.

Table S2

			Spectral count							
**Accession number**	**Protein**	**Mw (kDa)**	**Frac 23**	**Frac 24**	**Frac 25**	**Frac 26**	**Frac 27**	**Frac 28**	**Frac 29**	**Frac 30**
TMUE_s0015001100	Pancreatic alpha amylase	66		7	18	19	8	7		
TMUE_s0005004200	Malate dehydrogenase	39		3	5	17	9	3		4
TMUE_s0256000200	Hypothetical protein	70	3	4	7	13	12	12	8	6
TMUE_s0086000700	78 kDa glucose regulated protein	73	11	11	10	12	5		2	3
**TMUE_s0016004100**	**Serpin protein**	**177**	**3**	**3**	**7**	**12**	**2**	**5**		**3**
**TMUE_s0013010700**	**NADH dependent fumarate reductase**	**57**			**8**	**11**	**7**	**7**		
TMUE_s0055003300	Elongation factor 2	91				11	6	5		2
TMUE_s0084003500	CAP-domain containing protein	39	3	6	9	10	7	6		3
TMUE_s0058002100	Conserved hypothetical protein	26			4	9	7	6		4
TMUE_s0093000600	Protein disulfide isomerase A6	48		5	6	8	3	5	2	4
**TMUE_s0003017400**	**Rab GDP dissociation inhibitor alpha**	**48**			**6**	**8**	**5**	**4**		
**TMUE_s0016007600**	**Hypothetical protein**	**23**			**3**	**8**	**5**	**4**		**4**
TMUE_s0036003800	Parathyroid hormone 2 receptor	45	2	6	5	7	6	4		3
**TMUE_s0066001200**	**Ion trans 2 and Pfam-B 17708-domain containing protein**	**132**			**3**	**6**	**3**	**6**	**3**	**5**
TMUE_s0076003900	Trans sialidase	156	3		2	6	3	3		
TMUE_s0074001100	Hypothetical protein	53			4	6	3	5		2
TMUE_s0051004900	Sulfhydryl oxidase 1	70				6	5			2
TMUE_s0083000200	Inorganic pyrophosphatase	63		2	5	6	3			
**TMUE_s0009001300**	**Translationally controlled tumor protein**	**21**				**6**	**5**	**4**		**2**
TMUE_s0165000700	Cysteine glycine protein 2	12			3	5	4	3		5
TMUE_s0293000700	Hypothetical protein	23		2	3	5	4	5		5
TMUE_s0004017800	Porin	41			2	5	4	3	4	3
TMUE_s0106000600	Moesin:ezrin:radixin 1	69	4	2	4	5	3	3		2
TMUE_s0085005000	Venom allergen 5	39			3	5		2		3
**TMUE_s0146001900**	**Trypsin-domain containing protein**	**52**			**2**	**5**	**3**	**2**		
TMUE_s0072000900	ADP ribose pyrophosphatase, mitochondrial	29				5	2	3		
TMUE_s0012011300	Neurogenic locus notch protein 2	103		2	2	4	2	2		
TMUE_s0002013500	Conserved hypothetical protein	56			3	4	3			
**TMUE_s0072003400**	**Independent phosphoglycerate mutase**	**46**				**4**	**3**	**4**		
TMUE_s0098000400	Protein wos2	21				4		2		3
TMUE_s0217000400	Pfam-B 11092 and MBOAT-domain containing protein	269				4				
TMUE_s0104003200	NUC194 and PI3 PI4 kinase and FATC-domain containing protein	442	3			3				
TMUE_s0048003100	Vinculin	123		2	2	3				
TMUE_s0037007000	|Pfam-B_927_and_Beach_and_WD40-domain_containing_protein	369				3				
TMUE_s0241001300	Pkinase-domain containing protein	43				3				
TMUE_s0010008400	Disks large 5	208				3				
TMUE_s0019001900	DUF21-domain containing protein	63				3				
**TMUE_s0428000200**	**Lactoylglutathione lyase**	**20**				**3**		**2**		
**TMUE_s0132001200**	**Gut specific cysteine proteinase**	**39**				**3**	**2**			
TMUE_s0006005400	Autophagy protein 2 protein B	225				3				
TMUE_s0326000200	PAN 1-domain containing protein	34				3				
**TMUE_s0071003300**	**TPD52 domain containing protein**	**17**				**3**				
TMUE_s0175001100	E3 SUMO protein ligase RanBP2	235				2				
TMUE_s0189000300	Eukaryotic initiation factor 4A	50				2				
TMUE_s0145001100	Pfam-B 10329 and zf-CCHC-domain containing protein	57				2				
TMUE_s0004005500	Trypsin-domain containing protein	33				2				
TMUE_s0009011100	la protein	45				2			2	
TMUE_s0017002000	rve and Pfam-B 10329 and RVT 1 and Pfam-B 2707-domain containing protein	159				2				
TMUE_s0138001500	Arginine glutamic acid dipeptide repeats	148				2				
TMUE_s0004000500	Gut specific cysteine proteinase	48	2			2				
TMUE_s0013011900	Glyco_hydro_38_and_Alpha-mann_mid_and_Glyco_hydro_38C-domain_containing_protein	126				2				
TMUE_s0037005700	Thioredoxin	18				2				2
TMUE_s0005009900	Pfam-B 1842 and Pfam-B 3141 and tRNA int end N2 and uDENN and DENN and dDENN and Pfam-B 2124-domain containing pro	231				2				
TMUE_s0071006300	Pfam-B 16788-domain containing protein	123				2				
TMUE_s0049001100	Peptidase S9 prolyl oligopeptidase active site	150				2				
TMUE_s0011000700	Dsrm-domain containing protein	25				2				
TMUE_s0062003000	Nascent polypeptide associated complex protein	22				2				
TMUE_s0033004300	Probable nuclear transport factor nuclear transport factor	15				2				
TMUE_s0090002300	DEAD and Helicase C and dsRNA bind-domain containing protein	74				2				
TMUE_s0026000400	Alpha amylase	69				2				
TMUE_s0082003700	Pfam-B 2621-domain containing protein	34				2				
TMUE_s0304000500	Phenylalanine 4 hydroxylase	59				2				

Table S2. List of identified proteins with peak abundance around Superose 12 fractions 24 to 27. The protein content of Superose 12 fractions 23 to 32 was analysed by mass spectrometry. The total spectral count is displayed for each protein in each fraction (criteria set to 95% protein threshold, 50% peptide threshold, minimum 2 peptides identified). Proteins were sorted by spectral count in fraction 26 and those with peak abundance around fractions 24 to 27 are displayed here. Bold indicates proteins that were identified in both the Superose 12 and Superdex 75 chromatography steps. Mw= molecular weight in kDa. Frac = fraction.

Table S3

			Spectral count						
**Accession number**	**Protein**	**Mw (kDa)**	**Frac 17**	**Frac 18**	**Frac 19**	**Frac 20**	**Frac 21**	**Frac 22**	**Frac23**
TMUE_s0016011400	MULE and Pfam-B 516 and WAP-domain containing protein	71			50	68	77	67	13
TMUE_s000300760	WAP type 'four disulfide core'	41	6	11	34	45	46	37	15
TMUE_s0175001500	Porin	54	13	19	39	34	41	9	
TMUE_s0256000200	Hypothetical protein	70		2	5	16	27	10	7
TMUE_s0090001300	Porin	19				26	27	9	
TMUE_s0269000200	Triosephosphate isomerase	27		2	11	12	19	14	10
TMUE_s0191000800	Trypsin domain containing protein	79		3	8	13	17	4	
TMUE_s0256000600	Conserved hypothetical protein	48				7	17	7	4
TMUE_s0048003500	Serine protease	45			6	13	16	6	5
TMUE_s0103000900	Glutathion S transferase	23				3	14	4	2
TMUE_s0014006600	Motile sperm domain containing protein	15				7	12	9	5
**TMUE_s0016004100**	**Serpin**	**177**		**2**	**3**	**9**	**12**	**2**	
TMUE_s0033003400	Trypsin domain containing protein	37					12		
TMUE_s0023000300	Pfam-B 9093-domain containing protein	32			5	10	11	9	5
TMUE_s0208001600	Pathogenesis protein 1B	43	5	4	8	5	11	2	
**TMUE_s0003017400**	**Rab GDP dissociation inhibitor alpha**	**48**			**10**	**17**	**11**	**3**	**0**
TMUE_s0012009900	Hypothetical protein	45	2	4	7	5	10		
**TMUE_s0009001300**	**Translationally controlled tumour protein**	**21**				**2**	**10**	**4**	**3**
**TMUE_s0013010700**	**NADH dependent fumarate reductase**	**57**			**11**	**11**	**10**	**2**	
TMUE_s0177000800	Heat shock protein 70	71			6	9	9	4	
**TMUE_s0072003400**	**Independent phosphoglycerate mutase**	**46**			**2**	**7**	**9**	**2**	**2**
TMUE_s0078002100	Conserved hypothetical protein	41				2	9		
TMUE_s0047003900	Cap domain containing protein	58			4	4	8	2	
TMUE_s0006008700	Thioredoxin	22				3	8	2	
TMUE_s0031002800	Hypothetical protein	17					8	2	
TMUE_s0005016100	Peptidy proly cis trans isomerase FKBP4	52			2	3	8		
TMUE_s0042004400	Low density lipo receptor repeat	164			2	5	7	3	
TMUE_s0204000100	Motile sperm domain containing protein	21			4	3	7	3	2
TMUE_s0003007400	Peptidase, S1 subfamily	49			2		7	2	2
TMUE_s0023000500	Pfam-B 9093-domain containing protein	26			2	7	7	2	
TMUE_s0137001000	Hypothetical protein	43				5	7	5	
**TMUE_s0016007600**	**Hypothetical protein**	**23**				**7**	**7**	**2**	
TMUE_s0078004700	Copper:zinc superoxide dismutase	20					7		
TMUE_s0037003400	Pathogenesis protein 1B	41			2	6	6	4	2
TMUE_s0078001400	DUF290-domain containing protein	11			3	5	6		
TMUE_s0010006100	Calmodulin	18				5	6	3	
TMUE_s0007005500	Peroxiredoxin 2	121				2	6		
TMUE_s0085003400	Motile sperm domain containing protein	20					6		
TMUE_s0042005600	Motile sperm domain containing protein	24			4	5	5	3	
TMUE_s002200550	Eukaryotic elongation factor 1 delta	21			3	5	5	2	
TMUE_s0013012700	Motile sperm domain containing protein	14					5		
TMUE_s0074002600	Alcohol dehydrogenase NADP+ A	75					5		
TMUE_s0281000600	CBM 14 and TIL-domain containing protein	202					5		
**TMUE_s0132001200**	**Gut specific cystein proteinase**	**39**			**4**	**4**	**4**		
TMUE_s0004002000	Chymotrypsin inhibitor	16					4		2
TMUE_s0014013200	Heat shock protein 90	81				2	4	2	
TMUE_s0066002300	Hypothetical protein	16				4	4		
TMUE_s0133001900	Heat shocl 70 kDa protein 4	95			3	3	4		
TMUE_s0328000400	Hypothetical protein	19				4	4		
TMUE_s0004019100	Major sperm protein 1	14				2	4		
TMUE_s0038007500	DDE Tnp IS1595 and Pfam-B 9093-domain containing protein	25				3	3		
TMUE_s0003006100	Hypothetical protein	225				2	3		
TMUE_s0009000200	Pathogenesis protein 1B	19			3		3		
**TMUE_s0428000200**	**Lactoylglutathione lyase**	**20**				**2**	**3**		
TMUE_s0007006700	Chymotrypsin inhibitor	15					3		
TMUE_s0071006300	Pfam-B 16788-domain containing protein	123				2	3		
**TMUE_s0071003300**	**TPD52 domain containing protein**	**17**					**3**		
**TMUE_s0146001900**	**Trypsin domain containing protein**	**52**					**3**		
TMUE_s0074002400	Alcohol dehydrogenase NADP+ A	37					3		
**TMUE_s0066001200**	**Ion trans 2 and Pfam-B 17708-domain containing protein**	**132**					**3**		
TMUE_s0045002900	Serine protease inhibitor Kazal type 4	8					2		
TMUE_s0024004400	Conserved hypothetical protein	38					2		
TMUE_s0003007500	Coagulation factor IX	15					2		
TMUE_s0031002200	Uncharacterised transposase protein	39					2		
TMUE_s0136001600		63					2		
TMUE_s0009009900	Protein asteroid	28					2		
TMUE_s0208000600	WAP domain containing protein	25					2		
TMUE_s0005009800	Aminomethyltransferase, mitochondrial	91					2		
TMUE_s0024001300	TSP 1 and Reeler and Spond N and Peptidase M23 and Kunitz BPTI-domain containing prot	124					2		
TMUE_s0071007100	Cathepsin F	55					2		
TMUE_s0078001800	DUF290-domain containing protein	15					2		

Table S3. List of identified proteins with peak abundance around Superdex 75 fractions 20 to 22. The protein content of Superdex 75 fractions 17 to 23 was analysed by mass spectrometry. The total spectral count is displayed for each protein in each fraction (criteria set to 95% protein threshold, 50% peptide threshold, minimum 2 peptides identified). Proteins were sorted by spectral count in fraction 21 and those with peak abundance around fractions 20 to 22 are displayed here. Bold text indicates proteins that were identified in both the Superose 12 and Superdex 75 chromatography steps. Mw= molecular weight in kDa. Frac = fraction.
